# Computational studies of the binding profile of phosphoinositide PtdIns (3,4,5) P_3_ with the pleckstrin homology domain of an oomycete cellulose synthase

**DOI:** 10.1038/srep20555

**Published:** 2016-02-09

**Authors:** Guanglin Kuang, Vincent Bulone, Yaoquan Tu

**Affiliations:** 1 Division of Theoretical Chemistry and Biology, School of Biotechnology, Royal Institute of Technology (KTH), AlbaNova University Center, Stockholm, 106 91, Sweden; 2 Division of Glycoscience, School of Biotechnology, Royal Institute of Technology (KTH), AlbaNova University Center, Stockholm, 106 91, Sweden

## Abstract

*Saprolegnia monoica* is a model organism to investigate *Saprolegnia parasitica*, an important oomycete which causes considerable loss in aquaculture every year. *S. monoica* contains cellulose synthases vital for oomycete growth. However, the molecular mechanism of the cellulose biosynthesis process in the oomycete growth is still poorly understood. Some cellulose synthases of *S. monoica*, such as SmCesA2, are found to contain a plecsktrin homology (PH) domain, which is a protein module widely found in nature and known to bind to phosphoinositides, a class of signaling compounds involved in many biological processes. Understanding the molecular interactions between the PH domain and phosphoinositides would help to unravel the cellulose biosynthesis process of oomycetes. In this work, the binding profile of PtdIns (3,4,5) P_3_, a typical phosphoinositide, with SmCesA2-PH was studied by molecular docking, molecular dynamics and metadynamics simulations. PtdIns (3,4,5) P_3_ is found to bind at a specific site located at β1, β2 and β1-β2 loop of SmCesA2-PH. The high affinity of PtdIns (3,4,5) P_3_ to SmCesA2-PH is contributed by the free phosphate groups, which have electrostatic and hydrogen-bond interactions with Lys88, Lys100 and Arg102 in the binding site.

Some members of the oomycete class are plant or animal pathogens which cause severe environmental damages and economic losses every year^1^. A typical example is *Saprolegnia parasitica* from the order *Saprolegniales*, which is one of the most devastating fish pathogens^1^. As the cell wall of oomycetes are composed mainly of cellulose, the cellulose synthases of *S. parasitica* are potential targets to treat *Saprolegniosis* (*S. parasitica* infection) because these type of enzymes are vital for the pathogen^2^,^3^. In 2009, the sequences of several cellulose synthases of *Saprolegnia monoica*, which is closely related to *S. parasitica*, were determined by Fugelstad *et al*.^4^ These findings paved the way to investigate the structural basis of *Saprolegniosis* and to develop anti-*Saprolegniosis* drugs.

Among the sequences determined by Fugelstad *et al*., cellulose synthases 1, 2 and 4 are unique because they have a pleckstrin homology (PH) domain at the N-terminal. The PH domain is a 100–120 amino acid protein module which is widely spread in nature and can be found in a large number of proteins, from yeast to mammals^5^. The PH domain is known for its ability to bind phosphoinositides, which are important signaling compounds involved in signal transduction, membrane trafficking and other biophysical processes^6^. The phosphoinositide binding property has also been observed for the PH domain of cellulose synthase 2 of *S. monoica* (SmCesA2-PH)^7^. It has been determined that there are some pools of phosphoinositides and phosphoinositide kinases in oomycetes^7^. However, their functions have not been well characterized yet. The PH domain, which is a well-defined structural motif capable of binding to phosphoinositides with high affinity, might allow SmCesA2 to preferentially target some particular sites of the membrane. The phosphoinositide binding property of SmCesA2-PH is probably related to the regulation of the cellulose synthase activity and cell wall formation^7^. There are quite a few crystal structures available for the PH domains from other proteins with the soluble head groups of phosphoinositides^8^,^9^,^10^,^11^,^12^,^13^,^14^,^15^,^16^,^17^,^18^,^19^. These structures provide valuable insight into the binding of the PH domains with phosphoinositides. However, the detailed interactions between the PH domains and phosphoinositides in the membrane are poorly understood due to the complexity of protein-membrane systems. The PH domains of different proteins (pleckstrin^8^,^9^, β-spectrin^10^, phospholipase C-δ1^11^, Bruton’s tyrosine kinase^12^,^13^, dynamin^14^, DAPP1^15^ and TAPP1^16^) have very similar folding, namely a β-sandwich structure and a flanking α-helix (Fig. 1), though they have relatively low sequence identities, which usually range from 10–30%^5^. To date there is no structure available for the PH domain of any cellulose synthases of oomycetes. However, the conserved folding of PH domains makes homology modeling a feasible way to obtain a structure model of these unknown PH domains. In this work, a structure model of the PH domain of SmCesA2 was constructed by homology modeling, using the PH domain of human tandem PH-domain-containing protein 1 (TAPP1-PH)^16^ as the template. The binding profile of SmCesA2-PH with PtdIns (3,4,5) P_3_, a typical phosphoinositide^16^,^20^,^21^, was investigated in two steps. Firstly, the binding mode of the soluble inositol head groups of PtdIns (3,4,5) P_3_ with SmCesA2-PH was obtained by molecular docking, molecular dynamics and metadynamics simulations. Secondly, the binding profile of SmCesA2-PH with PtdIns (3,4,5) P_3_ in a POPC (1-palmitoyl-2-oleoyl-sn-glycero-3-phosphocholine) lipid bilayer was investigated based on the binding modes of the head groups obtained in step 1. The detailed interactions between SmCesA2-PH and PtdIns (3,4,5) P_3_/POPC were analyzed.

## Results and Discussion

### Sequence and Structure of SmCesA2-PH

**Figure 2:**
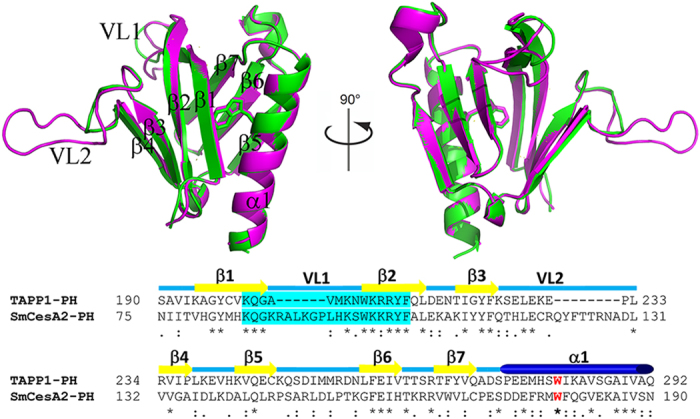
Sequence alignment and structure comparison of TAPP1-PH and SmCesA2-PH. The PPBM motifs are shaded in cyan and the invariant tryptophan is colored in red. The structures of TAPP1-PH and SmCesA2-PH are shown in the cartoon mode and are colored in green and pink, respectively.

The sequence identities between the PH domains from different proteins are very low (10% ~ 30%) in spite of their almost identical β-sandwich fold^5^. Through database searching, the PH domain of the human TAPP1 (TAPP1-PH) was selected as the template for homology modeling because it has the highest sequence identity (28%) with that of SmCesA2 (SmCesA2-PH). Figure 2 shows the sequences of TAPP1-PH and SmCesA2-PH aligned by Clustal Omega^22^. The amino acids of the two PH domains can be aligned very well, with no insertion or deletion in the β-sandwich and α-helix core structure. Only two insertions are found in the β1-β2 and β3-β4 loops of SmCesA2-PH. These two loops are least conserved in this class of protein domains and are usually called variable loop 1 (VL1) and variable loop 2 (VL2) since their sequences and structures are most variable^8^. There is a special section in the N-terminals (shaded in Fig. 2) of TAPP1-PH and SmCesA2-PH with the Lys-Xaa-Sma-Xaa_n_-Arg/Lys-Xaa-Arg-Hyd-Hyd motif (where ‘Xaa’ is any amino acid, ‘Sma’ is a small amino acid and ‘Hyd’ is a hydrophobic amino acid, n is a variable number). This motif is called the putative PtdIns (3,4,5)P_3_-trisphosphate-binding motif (PPBM) and is thought to be essential for the high affinity of the PH domains with PtdIns (3,4,5) P_3_^23^. The PPBM contains the C-terminal part of β1, VL1 and N-terminal part of β2. Interestingly, the two end points of the PPBMs of TAPP1-PH and SmCesA2-PH lying on β1 and β2, namely Lys-Xaa-Sma (KQG) and Arg/Lys-Xaa-Arg-Hyd-Hyd (KRRYF in TAPP1-PH and KKRYF in SmCesA2-PH), are almost identical. The only difference is the Xaa in the C-terminal endpoint, which is an arginine in TAPP1-PH but is a lysine in SmCesA2-PH. However, the variable Xaa_n_ part in VL1 is dramatically different in length and sequence identity, where SmCesA2-PH has 6 more residues. For TAPP1-PH, Ala203 of the Xaa_n_ part is reported to be critical to its specificity towards PtdIns (3,4) P_2_. When Ala203 is mutated to glycine, the specificity is lost and TAPP1-PH exhibits high affinity to both PtdIns (3,4) P_2_ and PtdIns (3,4,5) P_3_. The explanation given by Thomas *et al*.^16^ is that VL1 of TAPP1-PH is very short and therefore rather stiff. As a result, Ala203 has steric clashes with the 5-phosphate group of PtdIns (3,4,5) P_3_. Mutation to smaller glycine eliminates the steric clashes and improves the affinity for PtdIns (3,4,5) P_3_. As for SmCesA2-PH, the six additional residues on VL1 of SmCesA2-PH elongate VL1 and make it more flexible. Besides, Ala203 of TAPP1-PH is replaced by a lysine residue (Lys88) in SmCesA2-PH, which turns out to be important for the binding of PtdIns (3,4,5) P_3_ (this will be discussed in detail later). This might be the reason why SmCesA2-PH has high affinities to both PtdIns (3,4) P_2_ and PtdIns (3,4,5) P_3_^7^.

**Figure 3:**
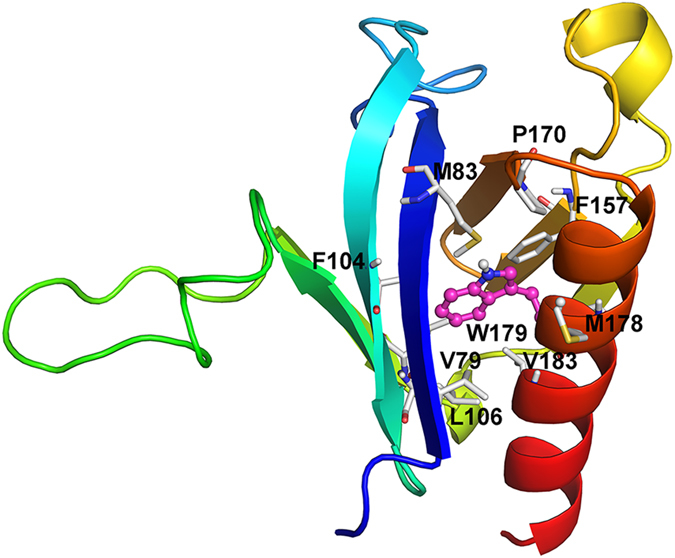
The hydrophobic cluster formed by Trp179 (in the ball-and-stick mode) and the residues from the β-sandwich structure (in the stick mode).

SmCesA2-PH has the same overall fold as the template TAPP1-PH, both having an antiparallel β-sandwich structure with two nearly orthogonal β-sheets and a flanking α-helix (Fig. 2). For SmCesA2-PH, residues 78–135 form the first four-stranded β-sheet which packs almost orthogonally against the second three-stranded β-sheet formed by residues 142–169. The C-terminal α-helix (residues 173–190) packs very closely with β1 of the first β-sheet and β5 and β6 of the second β-sheet and closes one corner of the β-sandwich structure (Fig. 2). Trp179 on the C-terminal α-helix is the only invariant residue in all the PH domains and is critical to their structures^14^. In the modeled SmCesA2-PH structure, Trp179 has the same conformation as its counterpart in the template. This residue, together with Val79, Met83, Phe104, Leu106, Ile111, Phe157, Pro170, Met178 and Val183, forms a compact hydrophobic cluster and brings the two β-sheets together to stabilize the β-sandwich structure (Fig. 3).

**Figure 4:**
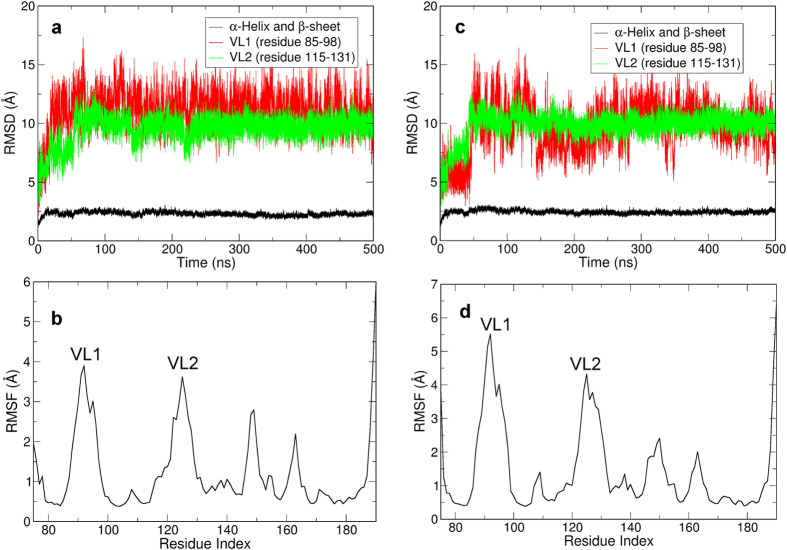
Dynamic behavior of SmCesA2-PH in two independent MD simulations. (**a** and **c**) RMSD plots of the backbone atoms of the β-sandwich and α-helix core structure (black), VL1 (red) and VL2 (green) of SmCesA2-PH. (**b** and **d**) The RMSF plot of SmCesA2-PH. (**a**) and (**b**) are the results of one simulation, while (**c**) and (**d**) are those of the other one.

PROCHECK^24^ was used to check the stereochemical quality of the structure model of SmCesA2-PH. The Ramachandran plot given by PROCHEK (Supplementary Fig. S1) shows that almost all the residues (93.3%) are in the most favored region, which proves the rationality of the SmCesA2-PH structure. In addition, MD simulations were carried out to relax the structure and check its dynamic stability in solution. The RMSD and RMSF plots in Fig. 4 show that the β-sandwich and α-helix core structure was rather stable in the MD simulations. Most fluctuations were observed in the loops, especially in VL1 and VL2. However, the loops didn’t have large scale flipping movements and were gradually stabilized as reflected by the RMSD plots (Fig. 4 and Supplementary Video S1). This optimized structure was used for subsequent docking studies. As a control, we also used the Amber99SB force field^25^ to validate the stability of the modeled structure and found that the structure has similar behaviors as with the CHARMM36 force field (Supplementary Fig. S2).

### Binding profile of SmCesA2-PH with the inositol head groups of PtdIns (3,4,5) P_3_

**Figure 5:**
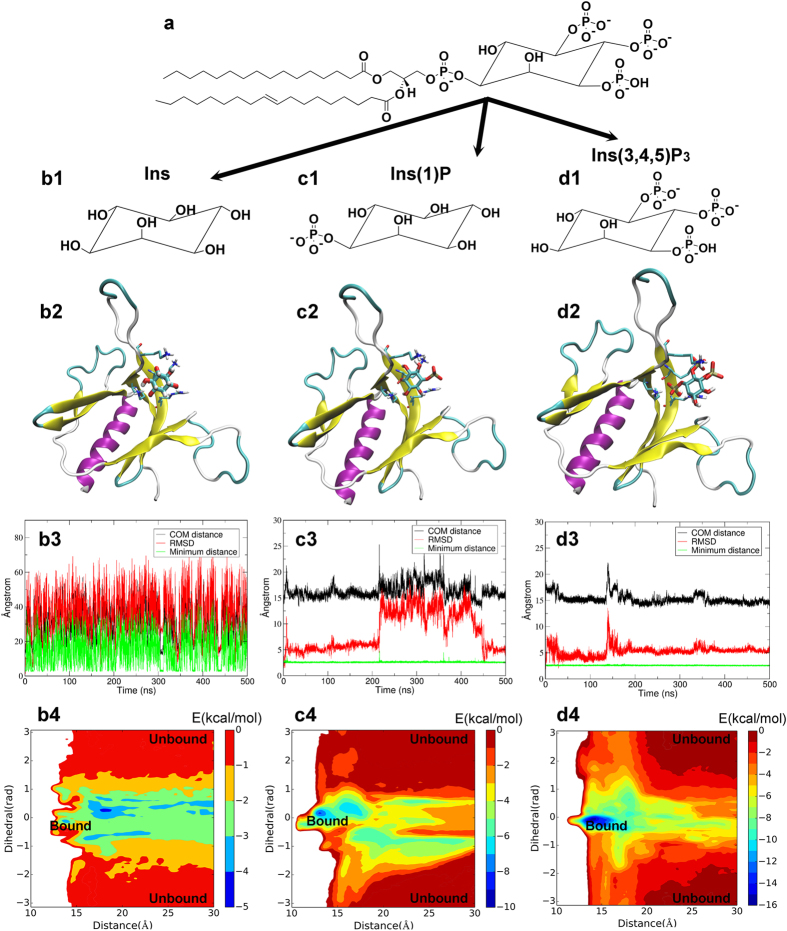
The binding profiles of Ins (b1-b4), Ins (1) P (c1-c4) and Ins (3,4,5) P_3_ (d1-d4) with SmCesA2-PH. b1, c1 and d1 are the structures of Ins, Ins (1) P and Ins (3,4,5) P_3_, respectively. b2, c2 and d2 show the binding modes of the head groups with SmCesA2-PH. b3, c3 and d3 are some MD plots of the head groups used to monitor their binding processes with SmCesA2-PH. COM distance is the distance between the centers of mass (COMs) of the head group and the protein. RMSD is the root mean square deviation of the head group conformation in MD simulation after alignment of the protein. Minimum distance is the minimum distance between the head group and protein heavy atoms. b4, c4 and d4 are the free energy surfaces of the head groups on the surface of SmCesA2-PH obtained by metadynamics.

The PH domain is best known for its high affinity with phosphoinositides, especially with PtdIns (3,4,5) P_3_ which is an important signaling compound involved in the membrane translocation process of the host protein of the PH domain^18^. Fugelstad *et al*. found that SmCesA2-PH binds with PtdIns (3,4,5) P_3_ with a strong intensity^7^. However, SmCesA2-PH is among the class of PH domains with poor specificity for PtdIns (3,4,5) P_3_ because it was also found to bind with other phosphoinositides with some observable intensities, which is most probably due to the additional basic residues in PPBM as discussed above. The only exception is the non-phosphorylated inositide (PtdIns), the binding of which to SmCesA2-PH was not observed^7^. Therefore, we believe that the free phosphate groups on the inositol ring of phosphoinositides are important for their binding to SmCesA2-PH, while the connecting phosphate group and the inositol ring likely have much lower binding affinity with SmCesA2-PH. In order to validate this, we investigated the binding profiles of SmCesA2-PH with different parts of the head group of PtdIns (3,4,5) P_3_, namely Ins, Ins (1) P and Ins (3,4,5) P_3_ (Fig. 5), using molecular docking, molecular dynamics and metadynamics simulations.

**Figure 6:**
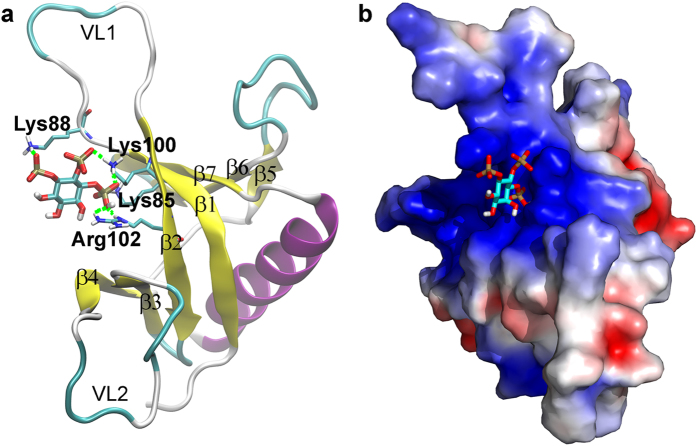
Binding profile of SmCesA2-PH with Ins (3,4,5) P_3_. (**a**) Binding mode of SmCesA2-PH with Ins (3,4,5) P_3_. (**b**) Electrostatic potential surface obtained with APBS^52^. Blue areas, +3 kT; red areas, −3 kT.

Molecular docking studies found that Ins, Ins (1) P and Ins (3,4,5) P_3_ can be docked into the same region of SmCesA2-PH formed by β1, β2 and VL1 (Figs 5b2,c2,d2 and 6a). This region corresponds to the center of the area with positive electrostatic potential (Fig. 6b). Therefore, the electrostatic interaction between SmCesA2-PH and Ins, Ins (1) P or Ins (3,4,5) P_3_ is most likely the major driving force for binding. When we carried out docking studies of Ins (3,4,5) P_3_ with the template structure TAPP1-PH, we found that it has a similar binding mode as with SmCesA2-PH (Supplementary Fig. S3). Several other PH domains have also been reported to have a similar binding site of phosphoinositols in this region^11^,^13^,^16^. However, the previous reported binding site also includes part of β3, β4 and VL2. This is because Ins (1,3,4,5) P_4_ was used in previous crystallization work and the 1-phosphate group would interact with the residues from β3, β4 and VL2. Similar to the previous results, the interaction between the basic residues and phosphate groups is the major type of interaction between SmCesA2-PH and the head groups of PtdIns (3,4,5) P_3_. This type of interaction is a combination of the electrostatic interaction and hydrogen-bond interaction and is termed the “salt-bridge” interaction in this work. For SmCesA2-PH, Lys85 from β1, Lys88 from the N-terminal of VL1 and Lys100 and Arg102 from β2 were found to be involved in the binding with the inositol head groups. All of the four basic residues are in the PPBM section of SmCesA2-PH (Fig. 2) and are called the “hot-spot” residues in this work. In addition, to avoid confusion, the states where the inositol head groups are bound to the hot-spot residues are called the “bound” states and the states where the inositol head groups have no interaction with any residue of SmCesA2-PH are called the “unbound” states. Besides, the states where no hot-spot residues are involved but the head groups are still attached to SmCesA2-PH are called the “contact” states, which can be deemed as the intermediary states between the bound and unbound states. Unlike the bound and unbound states, the contact states cannot be specifically marked on the free energy surface map and correspond to the vast area apart from the bound and unbound states (5b4,c4,d4).

For Ins, there is no salt-bridge interaction with the four hot-spot residues (Lys85, Lys88, Lys100 and Arg102) as it has no phosphate groups. Ins only has hydrogen-bond and van der Waals interactions with these residues. If the Ins head group is tightly bound to the protein, the three monitoring plots would be very stable, especially the minimum distance plot, as the two parts are in close contact. However, for Ins (Fig. 5b3 and Supplementary Fig. S4), the stability was only seen in the first several nanoseconds. After that, Ins dissociated from the protein and moved freely in the solution. Metadynamics is an enhanced sampling method which can construct the free-energy surface of a system using several specific collective variables^26^. As shown in Fig. 5b4, the free energy difference between the bound and unbound states of the SmCesA2-PH-Ins complex is so small (<5 kcal/mol) that Ins can bind to and unbind from SmCesA2-PH even in the unbiased MD simulation. Both MD simulation and metadynamics results demonstrate that the binding affinity between the inositol ring and SmCesA2-PH is very small.

The scenario of Ins (1) P is different as it is tightly attached to SmCesA2-PH during the whole MD simulation time, as reflected by the minimum distance curve (Fig. 5c3). However, there are significant fluctuations in the COM distance and RMSD plots (Fig. 5c3 and Supplementary Fig. S4). This is because Ins (1) P has just one phosphate group and can only have limited salt-bridge interaction with the hot-spot residues. Besides, there are several other basic residues (His84, Arg89, Lys92, His96, Lys97 and Lys101) spreading around the four hot-spot basic residues (Lys85, Lys88, Lys100 and Arg102). These basic residues can also have salt-bridge interactions with Ins (1) P in the simulation. The free energy difference (~10 kcal/mol) between the bound and unbound states is much higher than that in the Ins case, which is the reason why Ins (1) P stays attached to SmCesA2-PH in the MD simulation. However, the energy barriers of the bound state with respect to the stable contact states are rather small (<4 kal/mol). Therefore, Ins (1) P can drift away from the bound state to the contact states or *vice versa*. Our results reflect that the free phosphate group on the inositol ring has a high affinity to SmCesA2-PH. However, a single phosphate group is not enough to fix Ins (1) P in the hot-spot region. The results also indicate that the 1-phosphate group of Ins (1,3,4,5) P_4_ likely interferes with its binding to SmCesA2-PH as all the phosphate groups have high affinity with the basic residues of the protein.

Similar to Ins (1) P, Ins (3,4,5) P_3_ is also tightly attached to SmCesA2-PH, as observed from the MD simulation (Fig. 5d3). However, the fluctuations of the COM distance and RMSD plots of Ins (3,4,5) P_3_ are much smaller compared to Ins (1) P, which suggests a stronger binding of Ins (3,4,5) P_3_ with SmCesA2-PH. Ins (3,4,5) P_3_ sits in the positive potential center of SmCesA2 (Fig. 6b) and its three phosphate groups form salt-bridges with the hot-spot basic residues of SmCesA2-PH. These salt-bridge interactions tightly anchor Ins (3,4,5) P_3_ in the hot-spot region (Fig. 6a). The binding affinity in the hot-spot region (the bound state) is much higher than that in the contact states (Fig. 5d4), which makes Ins (3,4,5) P_3_ hard to move around dramatically as Ins (1) P in the MD simulation. The binding free energy in the hot-spot region is about −16 kcal/mol, which is rather high and is consistent with the high binding intensity observed in experiment^7^. In order to check the importance of the four hot-spot basic residues (Lys85, Lys88, Lys100 and Arg102), they were mutated to alanines in a control study. Schrödinger Glide XP docking^27^ was used for the redocking studies of Ins (3,4,5) P_3_ with MD-optimized SmCesA2-PH as well as the mutated forms. The docking scores are presented in Supplementary Table S1, which shows that the mutation of the hot-spot residues reduces the binding affinity of Ins (3,4,5) P3 to SmCesA2-PH significantly. This result further highlights the importance of these residues for the binding of phosphoinositides with the PH domain. To validate the stability of the meta-stable states, we selected a complex structure from the metadynamics simulation where Ins (3,4,5) P_3_ lies outside the hot-spot region (Supplementary Fig. S5). This state is selected because a similar binding mode with a minor population was observed in molecular docking studies. This state (with the COM distance ***d*** ~ 21.2 Å and the dihedral ϕ ~ 0.59 rad) which corresponds to the metastable cyan region in Fig. 5d4 has the energy of about 6 kcal/mol higher than that in the hot-spot region. In this state, the binding of Ins (3,4,5) P_3_ with SmCesA2-PH is stabilized by the salt-bridge interactions formed between Ins (3,4,5) P_3_ and Lys108 and Lys185 (Supplementary Fig. S5). However, this binding mode is unstable as observed in the MD simulations (Supplementary Fig. S5). In one simulation run, Ins (3,4,5) P_3_ moved close to the hot-spot region (with the COM distance ***d*** 14 ~ 16 Å and the dihedral ϕ ~ 0 rad) after about 335 ns. This observation also indicates that the hot-spot region is energetically more favorable for the binding of Ins (3,4,5) P_3_ with SmCesA2-PH.

The binding profiles of Ins, Ins (1) P and Ins (3,4,5) P_3_ show that the inositol ring itself has a very low binding affinity to SmCesA2-PH and the free phosphate groups on the ring are critical to their binding to SmCesA2-PH. One interesting issue is that PtdIns also has a connecting phosphate group between the inositol ring and the fatty acid tails. However, no adsorption is observed for PtdIns in experiment^7^. This is probably due to the spatial hindrance of the inositol ring which prevents this phosphate group from interacting with the protein. This issue is addressed in the following membrane adsorption section. Besides, we have carried out molecular docking and molecular dynamics simulations of SmCesA2-PH with a PtdIns (3,4,5) P_3_ molecule in solution. It is found that even though the two hydrophobic tails of PtdIns (3,4,5) P_3_ could not bind stably with SmCesA2-PH in the MD simulations, the inositol head group of PtdIns (3,4,5) P_3_ was bound tightly to the same site of SmCesA2-PH as the standalone head group stated above (Supplementary Fig. S6 and Supplementary Video S2). This result indicates that SmCesA2-PH is able to sequester phosphoinositides in solution.

### Binding profile of SmCesA2-PH with PtdIns (3,4,5) P_3_ in a POPC lipid bilayer.

Although there is a lot of structural data about the binding of PH domains with soluble inositol head groups (especially Ins (1,3,4,5) P_3_^11^,^13^,^16^), much less is known about the nature of the membrane-associated complex due to the experimental limitations of membrane systems. Actually, the systems consisting of SmCesA2-PH and the head groups of PtdIns (3,4,5) P_3_ discussed above are simplified models and cannot reflect the whole picture of the binding of SmCesA2-PH to PtdIns (3,4,5) P_3_ in the membrane due to structural and orientation restrictions of the bulky fatty acid tails. In this work, a POPC (1-palmitoyl-2-oleoyl-*sn*-glycero-3-phosphocholine) bilayer was used to mimic the membrane and a single PtdIns (3,4,5) P_3_ (POPIns (3,4,5) P_3_ is used in this work) molecule was inserted into the bilayer. SmCesA2-PH was placed on the top of the membrane with the hot-spot region facing the head group of PtdIns (3,4,5) P_3_ according to the procedures described in the method section. Two parallel 500 ns MD simulations with different initial velocities were carried out to equilibrate the SmCesA2-PH-PtdIns (3,4,5) P_3_/POPC complex. Similar phenomena were observed in the two simulations, where SmCesA2-PH was found to adsorb stably on the membrane rather than drifting around (Supplementary Fig. S7 and Supplementary Video S3). Besides, the binding of SmCesA2-PH to the PtdIns (3,4) P_2_/POPC membrane was also examined. As shown in Supplementary Fig. S8 and Supplementary Video S4, the binding profiles of SmCesA2-PH on the PtdIns (3,4) P_2_/POPC and PtdIns (3,4,5) P_3_/POPC membranes are rather similar, which reflects that SmCesA2-PH has similar affinities for PtdIns (3,4) P_2_ and PtdIns (3,4,5) P_3_. As a control, we have carried out simulations with pure POPC and PtdIns/POPC membranes. Although SmCesA2-PH can adsorb transiently on these two membranes for some time, most of the time it changes its position freely and is unable to bind tightly on the membranes (Supplementary Figs S9 and S10 and Supplementary Video S5 and S6). These results show that SmCesA2-PH has a low affinity to POPC or PtdIns.

**Figure 7:**
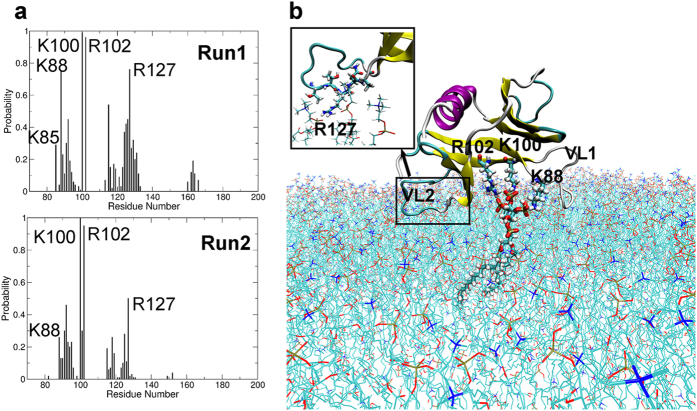
Binding profile of SmCesA2-PH with PtdIns (3,4,5) P_3_/POPC membrane. (**a**) Contact (within 3 Å) probability of the amino acids of SmCesA2-PH with the PtdIns (3,4,5) P_3_/POPC membrane. (**b**) Binding mode of SmCesA2-PH with membrane (Run1). The PtdIns (3,4,5) P_3_ molecule and the three hot-spot basic residues (Lys88, Lys100, Arg102) of SmCesA2-PH are shown in thick stick mode and the POPC molecules in thin stick mode. The protein is shown in the cartoon mode. The binding mode of VL2 with POPC molecules is shown in the inset.

The residues in contact (within 3 Å) with the PtdIns (3,4,5) P_3_/POPC membrane were counted in both simulations. Figure 7a shows that the residues having close contact with the membrane are Lys88, Lys100, Arg102 and Arg127. Lys88 from the N-terminal of VL1 and Lys100 and Arg102 from β2 can form salt-bridge interactions with the three free phosphate groups of PtdIns (3,4,5) P_3_ (Fig. 7b). This is consistent with the results of molecular docking and metadynamics discussed above. However, Lys85 from β1 doesn’t have much contact with PtdIns (3,4,5) P_3_ mainly due to distance restrictions, since the head group is only a small part of PtdIns (3,4,5) P_3_ and cannot adjust its conformation freely. Also, the linking phosphate group has little contact with SmCesA2-PH due to the distance and orientation restrictions, which explains the poor adsorption behavior of PtdIns in the experimental tests^7^. The high affinity of SmCesA2-PH to PtdIns (3,4,5) P_3_ is contributed mainly by the free phosphate groups on the inositol group. The consistency between the binding modes of SmCesA2-PH with a single soluble inositol head group and with the whole PtdIns (3,4,5) P_3_ lipid molecule in a membrane verifies the rationality of the initial orientation of SmCesA2-PH with respect to the PtdIns (3,4,5) P_3_/POPC membrane.

Besides Lys88, Lys100 and Arg102, which interact directly with PtdIns (3,4,5) P_3_, some residues on VL1 and VL2 were also found to have sporadic interactions with the POPC molecules of the membrane (Fig. 7a,b). However, the binding of VL1 or VL2 with the membrane is very unstable. This explains why dramatic conformational changes were observed for these two loops in the adsorption simulations (Supplementary Fig. S7). One prominent residue is Arg127, which forms salt-bridge interactions with the phosphate groups of POPC molecules and pulls VL2 towards the membrane (Fig. 7b). Other residues like Lys92, Gln115, Glu119, Gln122, Tyr123 and Thr125 also have electrostatic, van der Waals or hydrophobic interactions with the phosphate or choline groups of POPC. For example, the carboxylic group of Glu119 can have significant electrostatic interactions with the choline group of POPC when the distance is short. Besides, the nonpolar part of these residues can have hydrophobic interactions with the choline group which also has nonpolar methyl and ethylene groups. However, these interactions are not as stable as the salt-bridge interaction of Arg127 with the phosphate groups, as reflected by the probability plots in Fig. 7a. No penetration of the loops of SmCesA2-PH was seen in the MD simulation (Supplementary Video S3). This is because the surface of SmCesA2-PH is mainly composed of charged or polar residues (Fig. 6b), which makes it unable to penetrate into the interior of the membrane. As the cellulose synthase SmCesA2 is a transmembrane protein, the phosphoinositide binding property of the PH domain is probably related to the translocation of SmCesA2 in some specific parts of the cell. Indeed, the localization behavior of SmCesA2 in some sub-nuclear compartments has been observed experimentally^7^. Besides, it has been proposed that the activity of SmCesA2 can be regulated by binding to some specific phosphoinositides, which in turn is important for cell wall formation, a critical process for oomycete development^7^.

Lumb *et al*. have investigated the membrane-binding properties of GRP1-PH through molecular dynamics simulations^20^. Similar phenomena were observed, namely residues from the binding hot-spot region (β1, β2 and β1-β2 loop) bind tightly with PtdIns (3,4,5) P_3_ and VL1 (β1-β2 loop) and VL2 (β3-β4 loop) bind sporadically with POPC. Due to the sequence and structure differences between GRP1-PH and SmCesA2-PH, the residues responsible for binding are different. The most significant difference between their results and ours is in the β6/β7 loop. The β6/β7 loop of SmCesA2-PH is quite short (Fig. 2) and is actually quite far away from the membrane surface. However, the β6/β7 loop of GRP1-PH is much longer (28 resides) and contributes to the formation of the β-sandwich core structure of GRP1-PH. When GRP1-PH is bound to the POPC/PtdIns (3,4,5) P_3_ membrane, the β6/β7 loop can penetrate into the hydrophobic core of the membrane and stabilize the adsorption. The different binding profiles of the loops of SmCesA2-PH and GRP1-PH with PtdIns (3,4,5) P_3_ in the presence of the membrane are determined by the length and properties (polar and nonpolar residues) of the loops. Since the loops of the PH domains are the least conserved part of this structure module, the membrane binding profiles of different PH domains would be different.

## Conclusion

In this work, the structure of the PH domain of cellulose synthase 2 from *S. monoica* (SmCesA2-PH) was constructed by homology modeling using the PH domain of the human TAPP1 protein (TAPP1-PH) as the template. SmCesA2-PH has the same fold as TAPP1-PH, namely a β-sandwich structure plus a flanking α-helix. The inositol head groups of PtdIns (3,4,5) P_3_ were found to bind to SmCesA2-PH at a specific site consisting of residues from β1, β2 and β1-β2 loop (VL1). The inositol ring has a very low binding affinity with SmCesA2-PH. The high affinity of PtdIns (3,4,5) P_3_ to SmCesA2-PH is contributed by the free phosphate groups, which can have electrostatic and hydrogen-bond interactions with the basic residues in the binding site (Lys88, Lys100 and Arg102). The PtdIns (3,4,5) P_3_ molecule in a POPC bilayer has a similar binding mode to the soluble inositol head group Ins (3,4,5) P_3_. However, the inositol ring and the connecting phosphate don’t have much contact with SmCesA2-PH due to orientation and distance restrictions. Besides, the β1-β2 and β3-β4 loops may also contribute to the binding of SmCesA2-PH on the plasma membrane as sporadic interactions were seen with the POPC molecules. This work is the first one to investigate the PH domain from the carbohydrate synthases of oomycete at the atomistic level. We believe that our findings are helpful to understand the structure and mechanism of these carbohydrate synthases.

## Methods

### Preparation of the protein-ligand complexes

The sequence of the PH domain of SmCesA2 was extracted from the whole length sequence of SmCesA2 (Accession ID: C9WPJ9^4^). We found that the PH domain of the human TAPP1 (PDB code: 1EAZ^16^) has the highest sequence identity (28%) with SmCesA2-PH through the protein blasting utility of NCBI (http://blast.ncbi.nlm.nih.gov/). Therefore, this crystal structure was used as the template for homology modeling. Clustal Omega^22^ was then used to align the target and template sequences. Based on the aligned sequences, homology models of SmCesA2-PH were built using Modeller v9.11^28^. A total of 100 homology models were generated, and the model with the highest GA341 score^29^ and lowest Discrete Optimization Potential Energy (DOPE)^30^ was selected for subsequent analysis.

The selected homology model of SmCesA2-PH was placed in a cubic water box with the minimum distance of the protein atoms to the box edge set to 12.0 Å, which was then solvated with TIP3P water molecules^31^. 9 Cl^−^ ions were then added to neutralize the system. The CHARMM36 force field^32^ was used to model the protein and the protonation states of the amino acids were determined with PROPKA 3.0 (http://www.propka.org/)^33^.

The system thus obtained was first minimized for 1000 steps using the steepest descent algorithm. Thereafter, it was subject to two MD simulations for equilibrium. The first MD simulation was carried out for 100 ps at 300 K using a canonical isothermal-isochoric (NVT) ensemble and the second one was run for 500 ps using an isothermal-isobaric (NPT) ensemble with the pressure set to 1 atm and temperature to 300 K. In these two MD simulations, a harmonic potential with a force constant of 1000 kJ∙mol^−1^∙nm^−2^ was applied to constrain the protein heavy atoms. Finally, the system was subject to 500 ns MD simulations in an NPT ensemble with no position restraint on the protein. In all the MD simulations carried out in this work, a cut-off distance of 10 Å was used for short-range van der Waals and electrostatic interactions. The long-range electrostatic interactions beyond the cut-off were recovered by the Particle Mesh Ewald (PME) method^34^ with a 1.0 Å grid spacing. The LINCS algorithm^35^ was applied to constrain the bonds involving hydrogen atoms and a time step of 2 fs was used. The Gromacs 5.0.4 package (http://www.gromacs.org/), together with the external plugin PLUMED 2.1.1 36,37, was used to perform the MD simulations.

In this work, we investigated the binding modes of the inositol head groups of PtdIns (3,4,5) P_3_ with SmCesA2-PH. The structure of SmCesA2-PH optimized from MD simulations was used as the target for independent blind docking carried out by AUTODOCK 4.2 38. A grid box with a dimension of 56 × 56 × 56 Å^3^ was centered on the protein. This box was large enough to encompass the whole protein and leave sufficient space for the ligands to be docked to the protein surface. The Lamarckian Genetic Algorithm^39^ was used for conformational search of each ligand and was run for 100 times for the ligand, which generated 100 possible protein-ligand complexes (docking solutions). For each ligand, clustering of the docking solutions was conducted and the highest ranked protein-ligand complex from the largest cluster was selected.

The protein-ligand complexes of the three inositol head groups were first subjected to unbiased MD simulations to examine the stability of the complexes. The CHARMM general force field (v2b8) was used to model the head groups with the force field parameters generated by the ParaChem webserver (v0.9.7.1, https://cgenff.paramchem.org)^40^,^41^,^42^. The setup of the protein-ligand systems for the MD simulations was the same as that for the protein-only system mentioned above. The production runs were carried out for 500 ns using the isothermal-isobaric (NPT) ensemble at 1 atm and 300 K before running the metadynamics simulations.

### Metadynamics

Metadynamics is an enhanced conformation sampling approach where the sampling of a system is accelerated by introducing an additional biased potential with respect to some selected collective variables (CV)^43^ into the system. In metadynamics, the free energy surface (FES) of a system can be constructed as a function of collective variables. In this work, we used the well-tempered variant of metadynamics which can overcome the convergence problem of traditional metadynamics. In a well-tempered metadynamics simulation, the deposition rate of the biased potential decreases over the simulation time. At time *t*, the total biased potential *V* (*s*, *t*) added is given by:


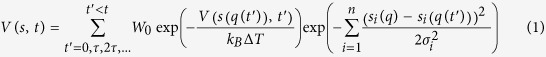


and the free energy *F* (*s*, *t*) as a function of the collective variables is determined by^44^,^45^


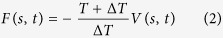


In equations (1) and (2), *s* presents the selected collective variables which are a function of the coordinates *q* of the system; τ is the Gaussian deposition rate; *W*_*0*_ is the initial Gaussian height; *k*_*B*_ is the Boltzmann constant; *T* is the temperature of the simulation; Δ*T* is an input parameter with the dimension of temperature; σ_*i*_ is the Gaussian width of the *i*th collective variable.

In this work, two collective variables were selected to describe the locations of the ligands relative to the protein (Supplementary Fig. S11) during the metadynamics simulations, which are (1) the distance ***d*** between the center of mass (COM) of the ligand and that of SmCesA2-PH; (2) the dihedral angle (torsion ϕ) defined by the two end points of the α-helix, the COM of the protein, and the COM of the ligand. The CVs were selected on a trial and error basis with the concern of stability and efficiency of the metadynamics simulations. Gaussians were added every 2 ps with the Gaussian widths of 0.3 Å and 0.05 rad for CV1 and CV2, respectively. The initial Gaussian heights were 0.1 kcal/mol for Ins and 0.5 kcal/mol for Ins (1) P and Ins (3,4,5) P_3_, respectively. ΔT was set to 600 K for Ins and 2100 K for Ins (1) P and Ins (3,4,5) P_3_, respectively. Since SmCesA2-PH has an irregular shape and two long loops, a 30 Å upper-wall constraint was applied to CV1 to limit the sampling of remote unbound regions and focus on the regions surrounding the β-sandwich and α-helix core structure. The regions on the tips of the loops are weak binding sites as these regions have only a few residues interacting with the inositol head groups. In this work, we found that it is inappropriate to use the distance as the only CV to describe different states because many conformations around the protein center have the same COM distance value. Therefore, we use the combination of distance (CV1) and dihedral (CV2) to discriminate different states, which are marked in Fig. 5. The metadynamics simulations were run until convergence (Supplementary Fig. S12), when the heights of Gaussian potential added were small. The helix content of SmCesA2-PH which was likely influenced by the metadynamics simulation was monitored with the ALPHARMSD (NN = 2, MM = 4, R_0 = 0.08) collective variable of PLUMED^46^. The stability of the helix (Supplementary Fig. S13) indicates that the CVs are good for the sampling of the binding poses of phosphoinositides on the surface of SmCesA2-PH. The results of metadynamics simulations were analyzed by a script written by Xianqian Sun, which is able to construct the free energy surfaces^47^.

### Adsorption on the membrane

POPC (1-palmitoyl-2-oleoyl-sn-glycero-3-phosphocholine) lipid bilayers have been widely used as a model for eukaryotic membranes^20^,^48^. In this work, a lipid bilayer composed of 249 POPC lipid molecules and 1 PtdIns (3,4,5) P_3_ (POPIns (3,4,5) P_3_ is used in this work) molecule was generated by CHARMM GUI (http://www.charmm-gui.org)^49^. This PtdIns (3,4,5) P_3_ molecule was then extracted and docked into the binding pocket of SmCesA2-PH identified by molecular docking and metadynamics. PtdIns (3,4,5) P_3_ was treated rigidly in this docking process to avoid conformational changes and make it easier to place SmCesA2-PH on top of the PtdIns (3,4,5) P_3_/POPC membrane. The grid box was centered on Lys85, Lys88, Lys100 and Arg102, with a dimension of 22.5 Å × 22.5 Å × 75 Å, which provides sufficient space for accommodating the two long tails of PtdIns (3,4,5) P_3_. Other docking parameters were the same as those for the inositol head groups as mentioned above. The dominant docked conformation of the SmCesA2-PH-PtdIns (3,4,5) P_3_ complex was then selected to align with the PtdIns (3,4,5) P_3_/POPC membrane. After alignment, SmCesA2-PH was moved upwards 10 Å along the z-axis to avoid overlap with the lipid molecules (Supplementary Fig. S14). The SmCesA2-PH-membrane complex thus obtained was then solvated with 21724 water molecules and neutralized with 3 Cl^−^. Similar to the previous MD simulations, the system also went through energy minimization (5000 steps), heating (100 ps), density equilibrating (10 ns) and production run (500 ns) steps. A harmonic potential with a force constant of 1000 kJ∙mol^−1^∙nm^−2^ was applied to constrain the heavy atoms of SmCesA2-PH and PtdIns (3,4,5) P_3_ in the heating and density equilibrating steps. Two parallel simulations with different initial velocities were carried out. As a control, we simulated the adsorption of SmCesA2-PH on POPC, PtdIns/POPC and PtdIns (3,4) P_2_/POPC membranes by replacing the PtdIns (3,4,5) P_3_ molecule of the PtdIns (3,4,5) P_3_/POPC membrane with POPC, PtdIns and PtdIns (3,4,5) P_3_ molecules, respectively. The same simulation setup and procedures were used for these two systems.

## Additional Information

**How to cite this article**: Kuang, G. *et al*. Computational studies of the binding profile of phosphoinositide PtdIns (3,4,5) P_3_ with the pleckstrin homology domain of an oomycete cellulose synthase. *Sci. Rep*. **6**, 20555; doi: 10.1038/srep20555 (2016).

## Supplementary Material

Supplementary Information

Supplementary Movie S1

Supplementary Movie S2

Supplementary Movie S3

Supplementary Movie S4

Supplementary Movie S5

Supplementary Movie S6

## Figures and Tables

**Figure 1:**
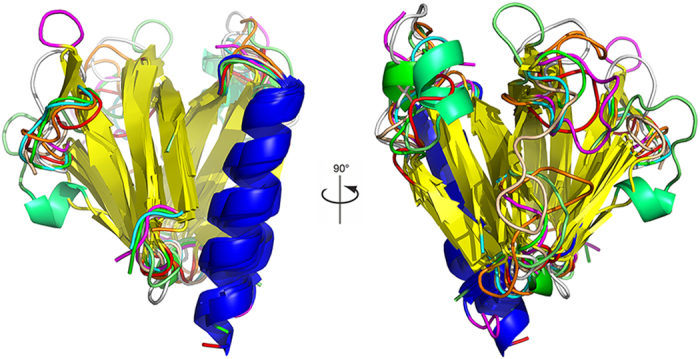
The common fold of the PH domains from different proteins (pleckstrin, β-spectrin, phospholipase C-δ1, Bruton’s tyrosine kinase, dynamin, DAPP1 and TAPP1). The β-sandwich structure is colored in yellow and the flanking α-helix in blue. The molecular graphics in this work were produced using PyMol v1.3^50^ and Visual Molecular Dynamics (VMD) v1.91^51^.
