# Supplementary material for: Computational studies of the binding profile of phosphoinositide PtdIns (3,4,5) P3 with the pleckstrin homology domain of an oomycete cellulose synthase
Source: Sci Rep. 2016 Feb 9;6:20555. doi: 10.1038/srep20555 (PMC4746654; doi:10.1038/srep20555)
Supplement: Supplementary Information [file srep20555-s1.pdf]

Supplementary Material

Computational studies of the binding profile of phosphoinositide PtdIns(3,4,5)P_3 with the pleckstrin homology domain of an oomycete cellulose synthase

Guanglin Kuang[1], Vincent Bulone[2], and Yaoquan Tu[1,*]

[1] Division of Theoretical Chemistry and Biology, School of Biotechnology, Royal Institute of Technology (KTH), AlbaNova University Center, Stockholm, 10691, Sweden

[2] Division of Glycoscience, School of Biotechnology, Royal Institute of Technology (KTH), AlbaNova University Center, Stockholm, 106 91, Sweden

* E-mail: yaoquan@kth.se

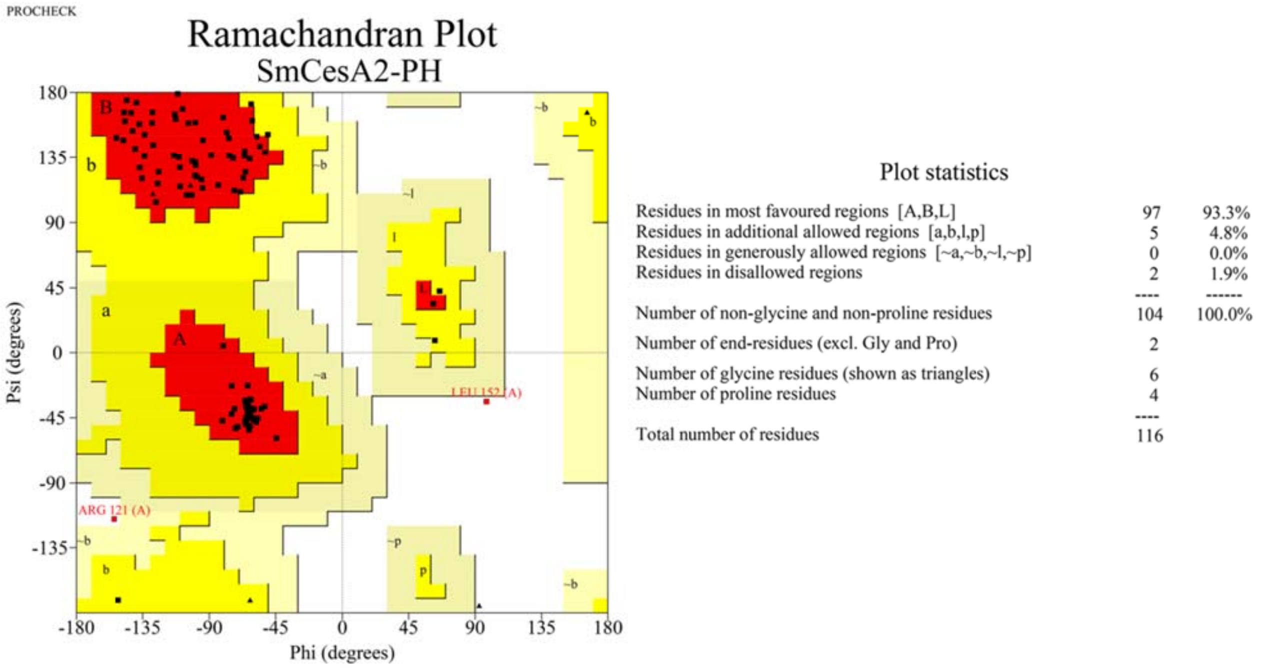

Supplementary Figure S1. Ramachandran plot of the homology model of SmCesA2-PH.

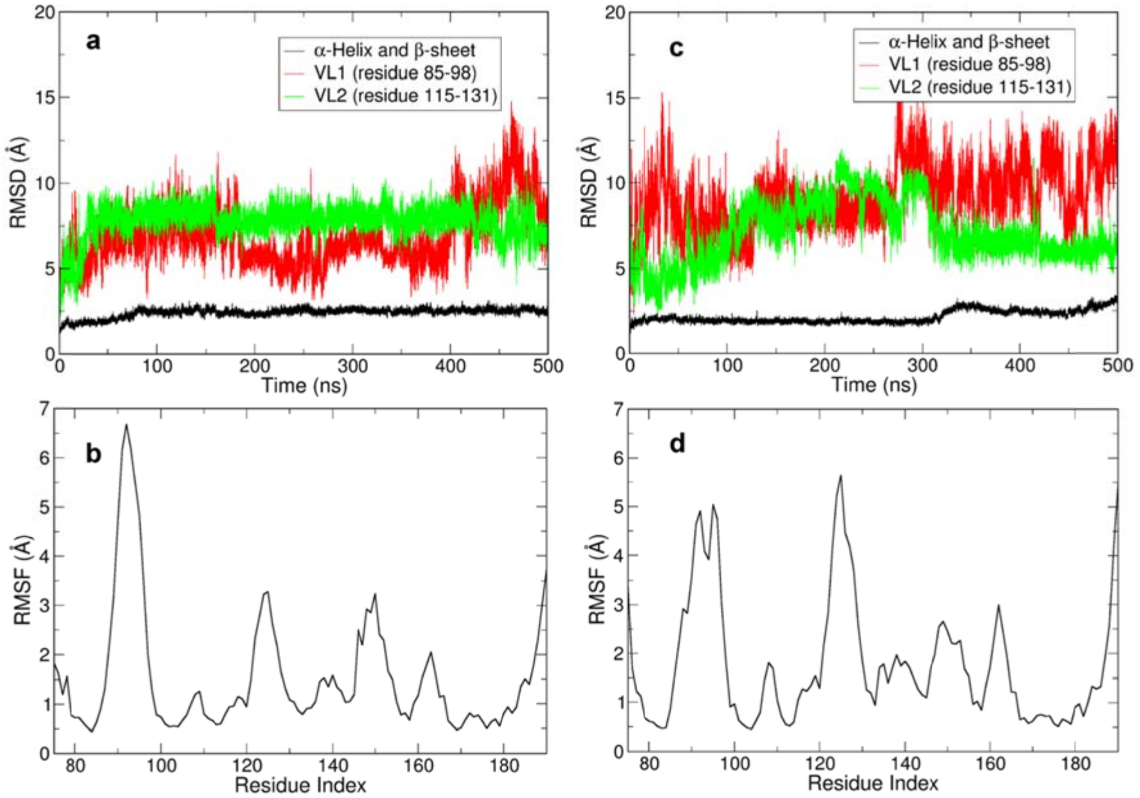

Supplementary Figure S2. Dynamic behaviors of SmCesA2-PH in two independent MD simulations with the Amber99SB force field. (a, c) RMSD plots of the backbone atoms of the β-sandwich and α-helix core structure (black), VL1 (red) and VL2 (green) of SmCesA2-PH. (b, d) The RMSF plot of SmCesA2-PH.

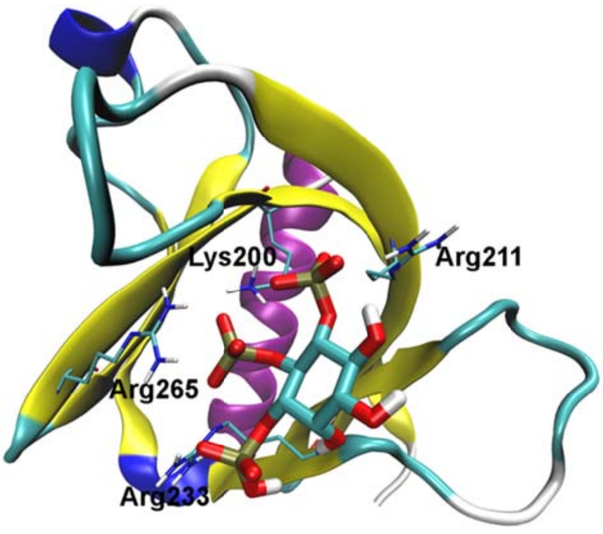

Supplementary Figure S3. Binding mode of Ins(3,4,5)P_3 with the template structure TAPP1-PH.

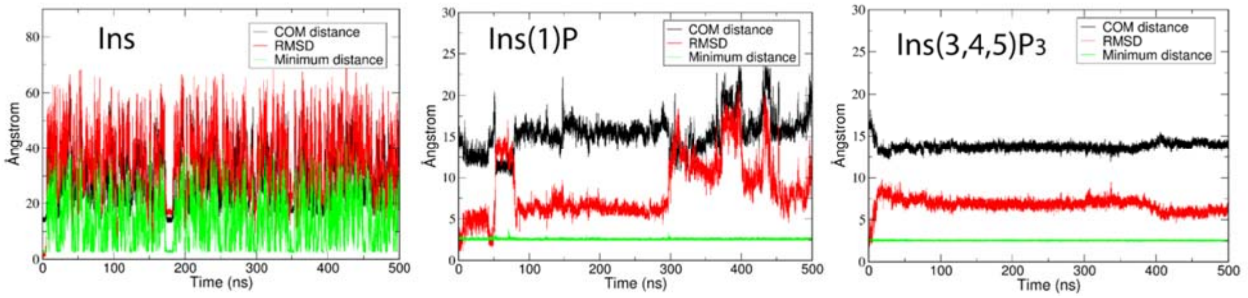

Supplementary Figure S4. Some MD plots of the head groups used to monitor their binding processes with SmCesA2-PH in the MD simulations with different initial velocities (second run). COM distance is the distance between the centers of mass (COMs) of the head group and the protein. RMSD is the root mean square deviation of the head group conformation in the MD simulations after the alignment of the protein. Minimum distance is the minimum distance between the head group and protein heavy atoms.

Supplementary Table S1. Schrödinger XP docking scores of Ins(3,4,5)P_3 with the mutated forms of the hot-spot residues identified by molecular docking and molecular dynamics simulations.

	Glide XP Docking Score (kcal/mol)
wild type	-7.57
K85A	-7.03
K88A	-7.00
K100A	-6.75
R102A	-7.27

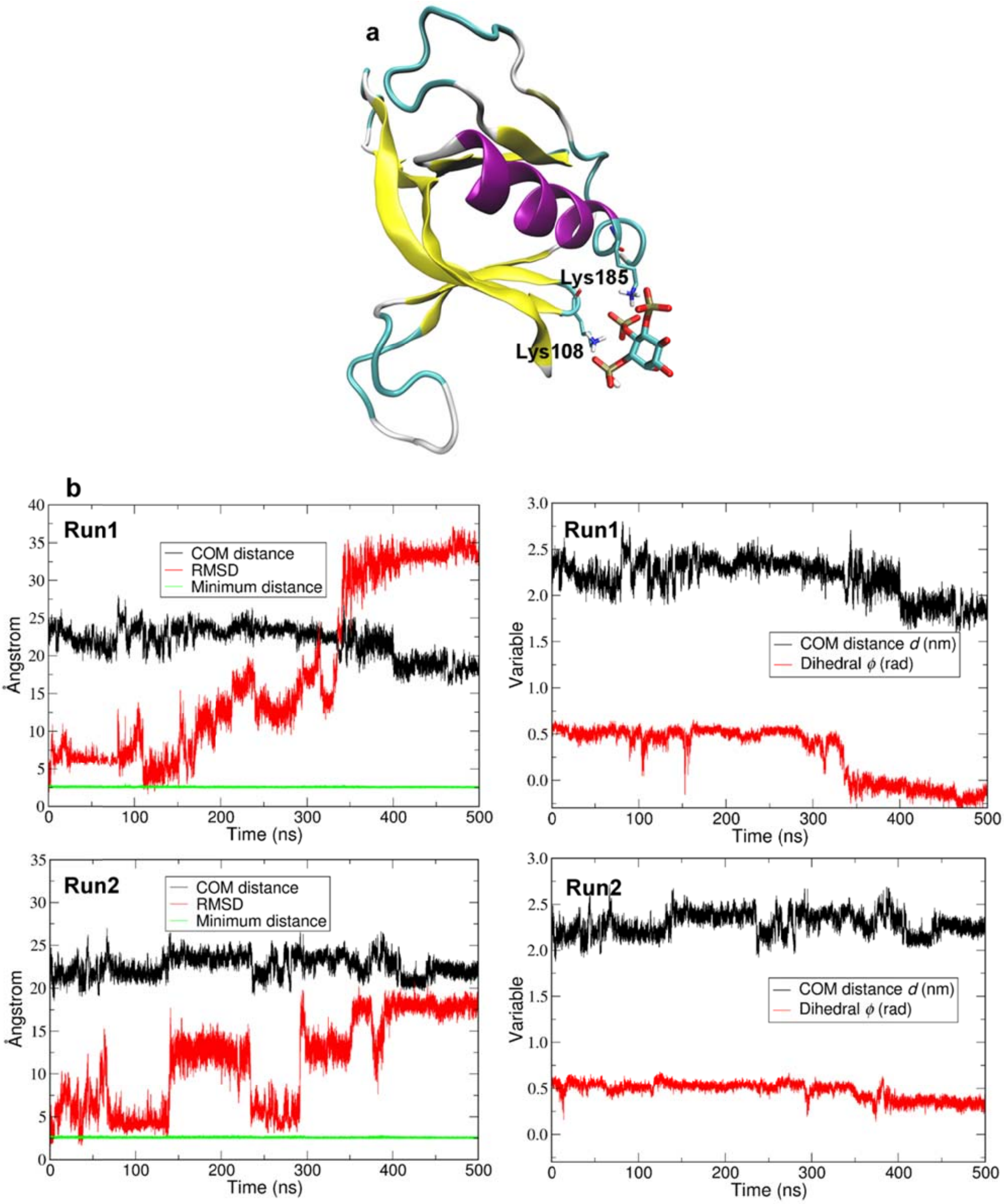

Supplementary Figure S5. (a) Binding mode of Ins(3,4,5)P_3 in a meta-stable state.(b) Some parameters to monitor the binding profile of the meta-stable state. COM distance is the distance between the centers of mass (COMs) of the inositol head group and the protein. RMSD is the root mean square deviation of the head group conformation in the MD simulation after the alignment of the protein. Minimum distance is the minimum distance between the head group and protein heavy atoms. Dihedral ϕ is the same as that defined in the Method section.

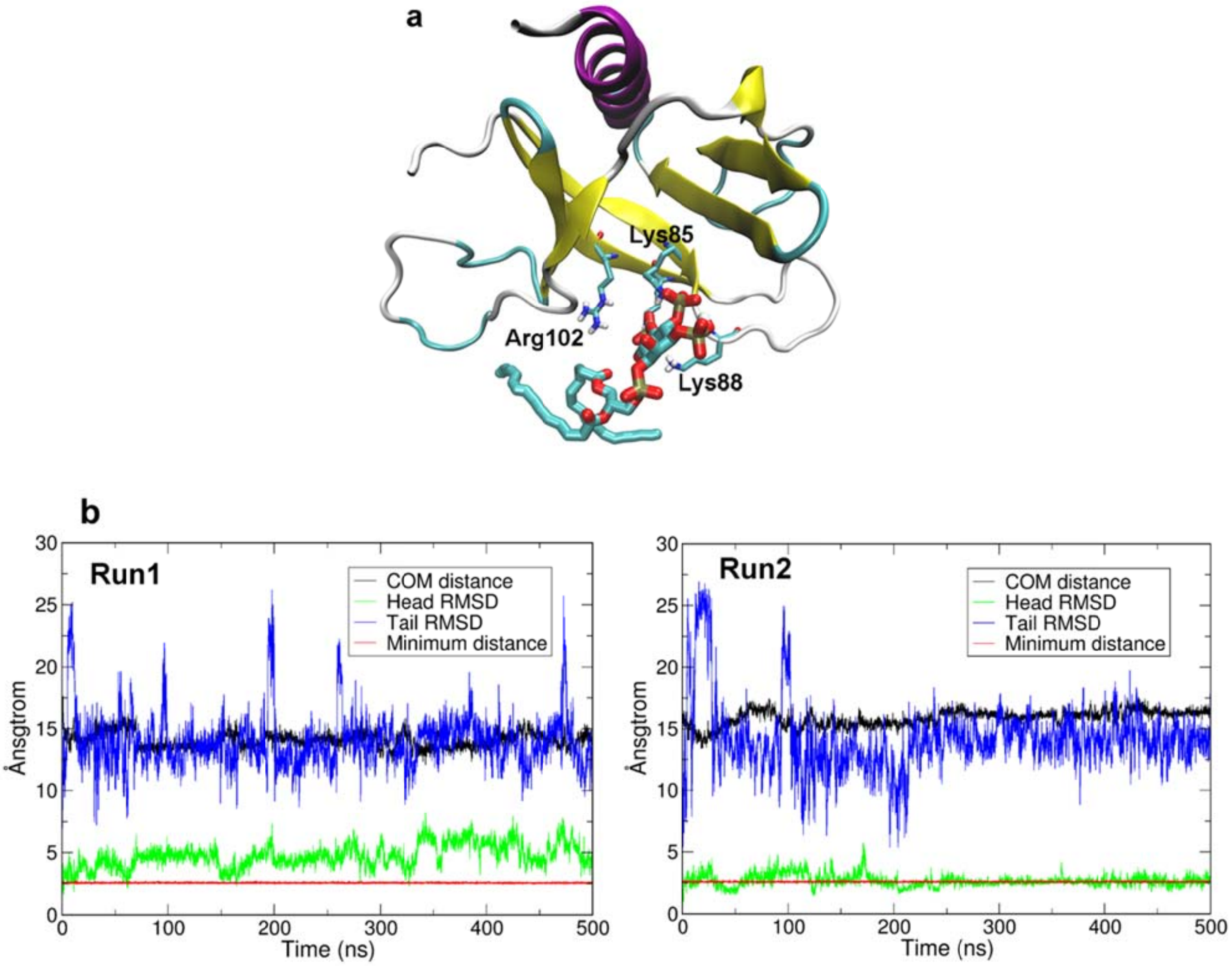

Supplementary Figure S6. (a) Binding mode of SmCesA2-PH with the PtdIns(3,4,5)P_3 molecule in solution. (b) Some parameters used to monitor the binding profile in the MD simulations. COM distance is the distance between the centers of mass (COMs) of the inositol head group and the protein. Head RMSD is the RMSD of the inositol head group. Tail RMSD is the RMSD of the two hydrophobic tails. Minimum distance is the minimum distance between the lipid molecule and protein heavy atoms.

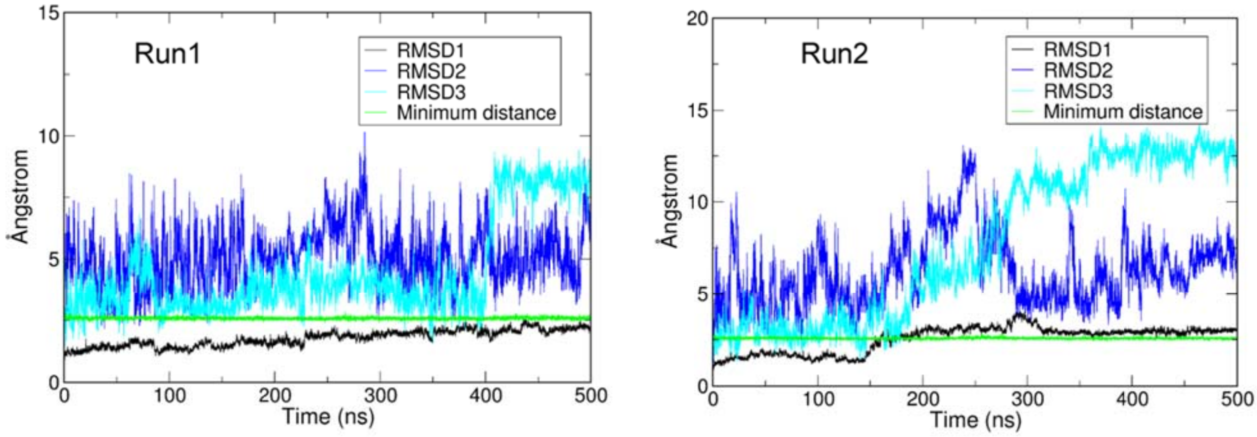

Supplementary Figure S7. **Binding profile of SmCesA2-PH on the PtdIns(3,4,5)P_3/POPC membrane in two independent MD simulations.** The figures show the plots of the RMSDs of the backbone atoms of the β-sandwich and α-helix core structure (RMSD1, black), VL1 (RMSD2, blue) and VL2 (RMSD3, cyan) as well as the minimum distance between SmCesA2-PH and the PtdIns(3,4,5)P_3/POPC membrane (green).

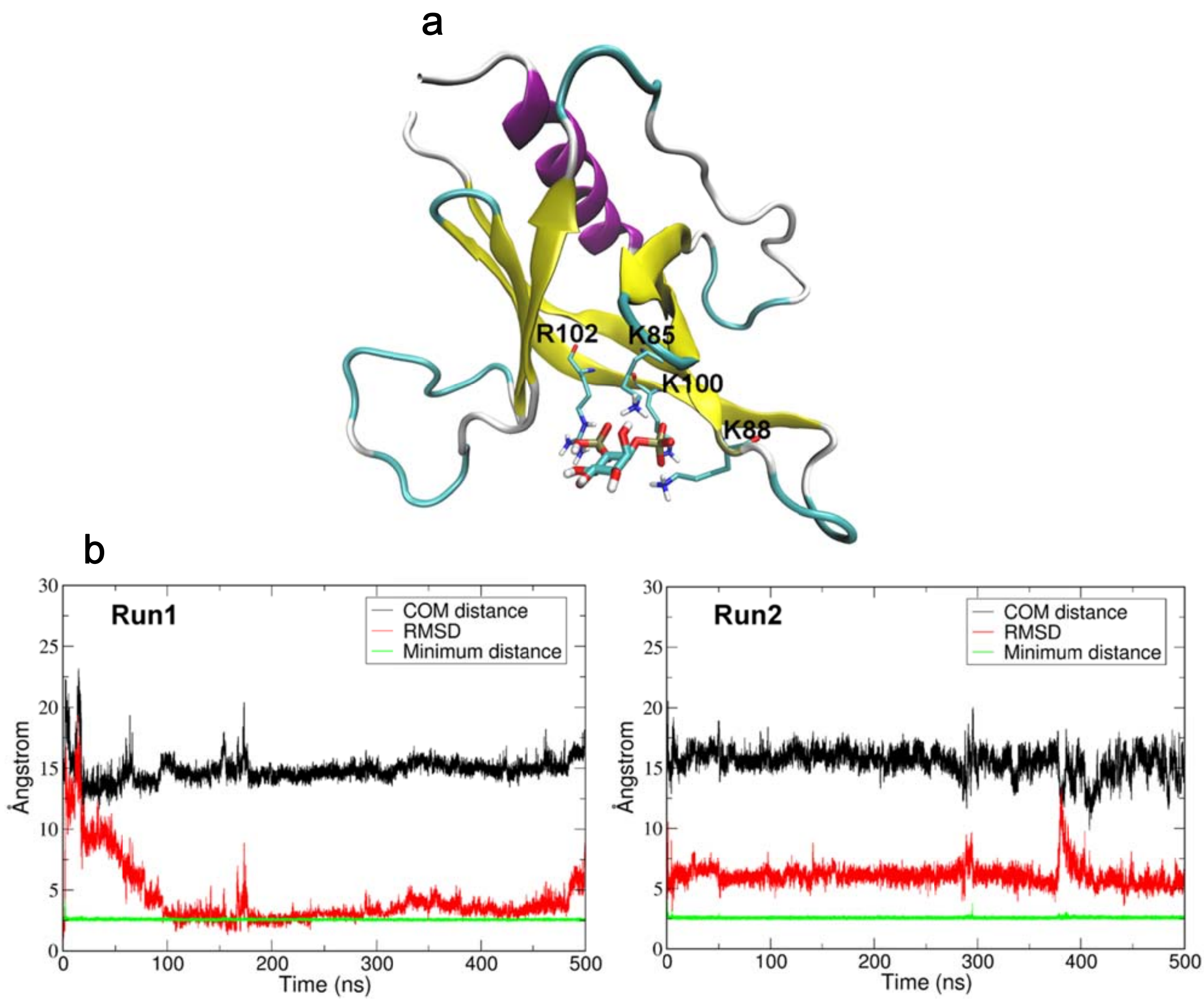

c

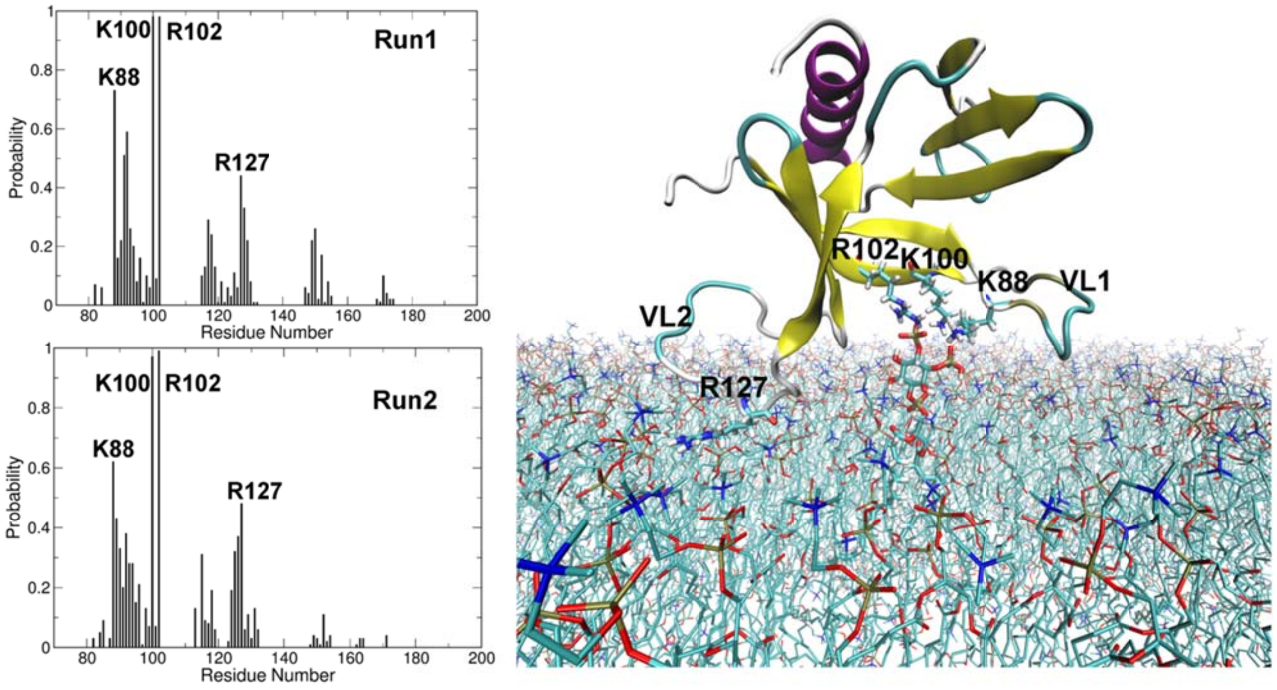

d

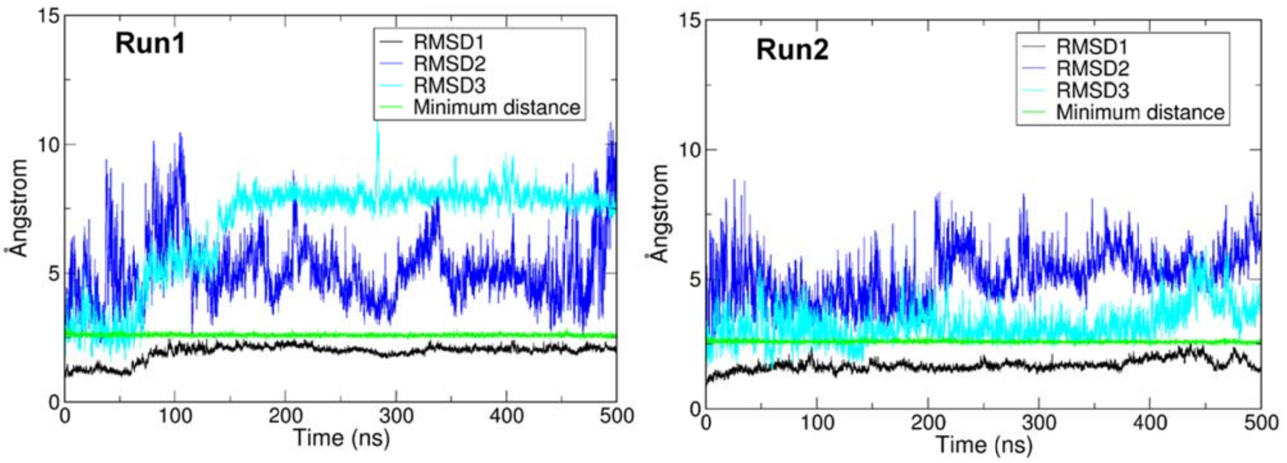

Supplementary Figure S8. Binding profile of SmCesA2-PH with PtdIns(3,4)P_2. (a) The binding mode of SmCesA2-PH with the head group Ins(3,4)P_2. (b) Some parameters used to monitor the binding profile of the head group in MD simulations. COM distance is the distance between the centers of mass (COMs) of the inositol head group and the protein. RMSD is the root mean square deviation of the head group conformation in the MD simulation after the alignment of the protein. Minimum distance is the minimum distance between the head group and protein heavy atoms. (c) The binding profile of SmCesA2-PH with the PtdIns(3,4)P_2/POPC membrane which includes the contact (within 3 Å) probability plots of the amino acids of SmCesA2-PH with the PtdIns(3,4)P_2/POPC membrane, and the binding mode of SmCesA2-PH with the membrane. (d)

The figures showing the plots of the RMSDs of the backbone atoms of the β-sandwich and α-helix core structure (RMSD1, black), VL1 (RMSD2, blue) and VL2 (RMSD3, cyan) as well as the minimum distance between SmCesA2-PH and the PtdIns(3,4)P_2/POPC membrane (green).

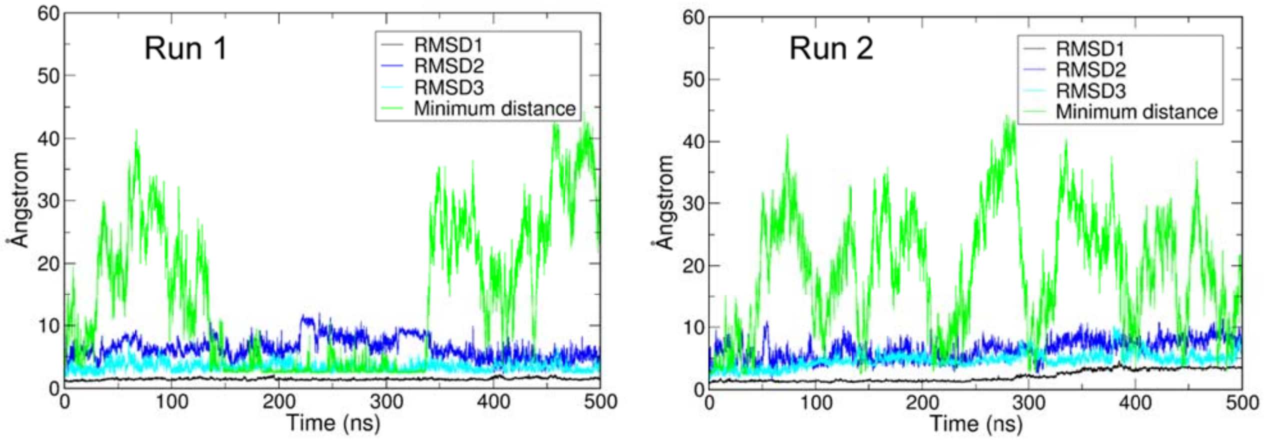

Supplementary Figure S9. **Binding profile of SmCesA2-PH on the POPC membrane in two independent MD simulations.** The figures show the plots of the RMSDs of the backbone atoms of the β-sandwich and α-helix core structure (RMSD1, black), VL1 (RMSD2, blue) and VL2 (RMSD3, cyan) as well as the minimum distance between SmCesA2-PH and the POPC membrane (green).

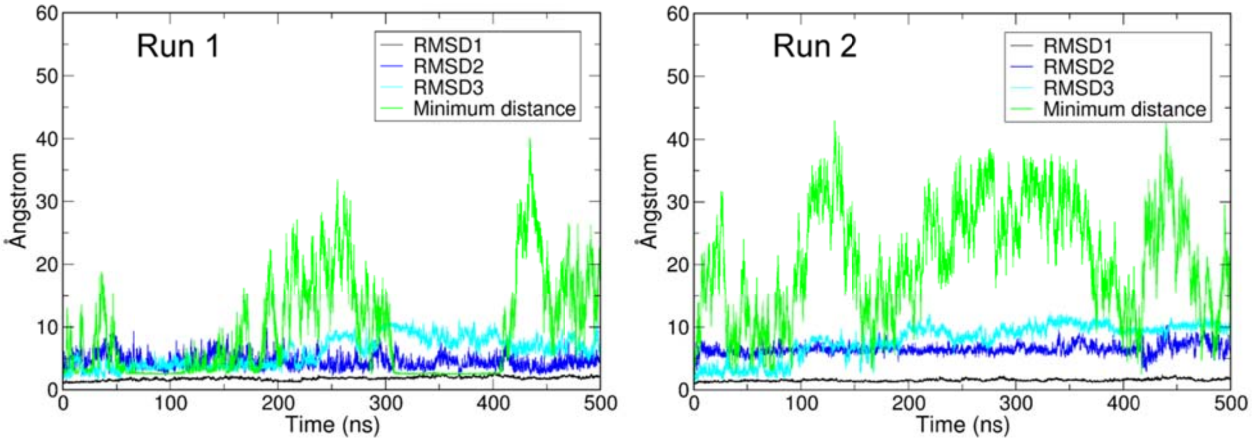

Supplementary Figure S10. **Binding profile of SmCesA2-PH on the PtdIns/POPC membrane in two independent MD simulations.** The figures show the plots of the RMSDs of the backbone atoms of the β-sandwich and α-helix core structure (RMSD1, black), VL1 (RMSD2, blue) and VL2 (RMSD3, cyan) as well as the minimum distance between SmCesA2-PH and the PtdIns/POPC membrane.

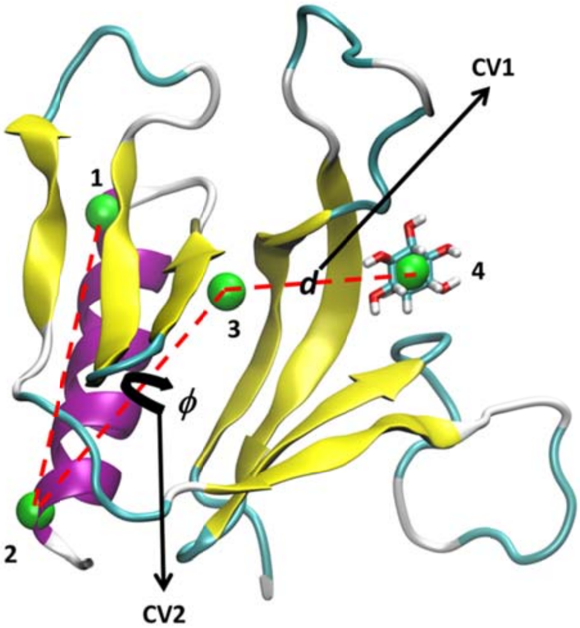

Supplementary Figure S11. **Illustration of the collective variables selected for the metadynamics simulations of the SmCesA2-Ins complex**. CV1 is the distance ***d*** between the center of mass (COM) of the ligand and that of SmCesA2-PH. CV2 is the dihedral angle (torsion ***ϕ***) defined by the two end points of the α-helix, the COM of the protein, and the COM of the ligand.

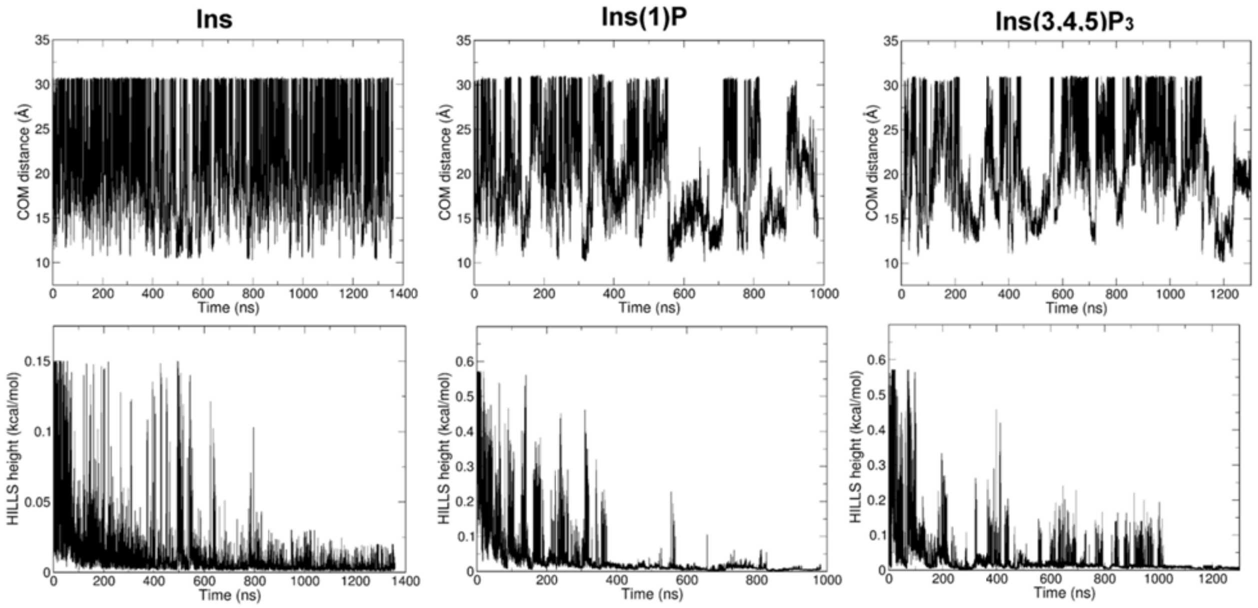

Supplementary Figure S12. Evolutions of COM distance (CV1) and Gaussian HILLS height in the metadynamics simulations. Several recrossing events of the distance CV and small Gaussian HILLS height (close to zero) are two common indicators of the convergence of the

metadynamics simulations. These plots indicate that the three systems have converged.

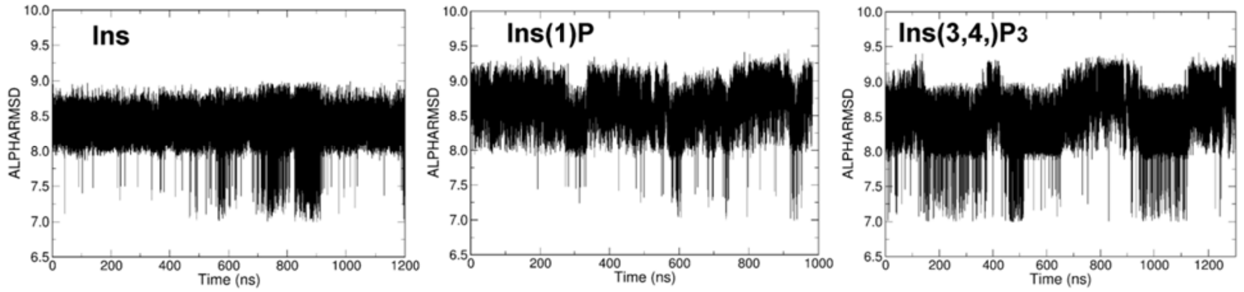

Supplementary Figure S13. Evolutions of the ALPHARMSD in the three metadynamics simulations, which can be used to monitor the content of α-helix.

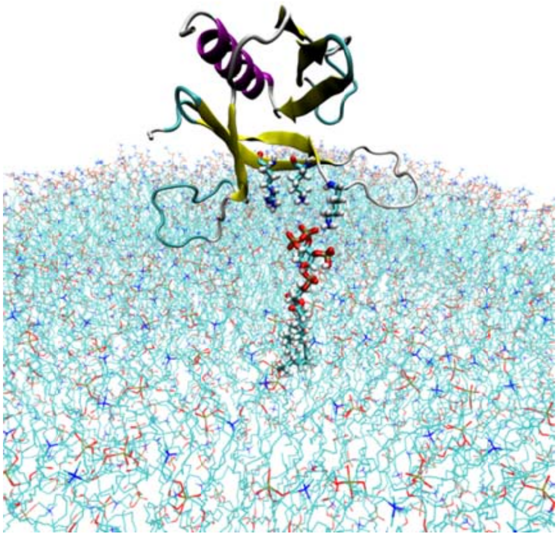

Supplementary Figure S14. Initial setup of the SmCesA2-PH and PtdIns(3,4,5)P_3/POPC membrane system. The PtdIns(3,4,5)P_3 molecule and three critical residues in the binding site of SmCesA2-PH are shown in the thick stick mode and the POPC molecules in the thin stick mode. The protein is shown in the cartoon mode.

Supplementary Videos

Supplementary Video S1. Trajectory of SmCesA2-PH in a 500 ns MD simulation.

Supplementary Video S2. Binding profile of SmCesA2-PH with the PtdIns(3,4,5)P_3 molecule in solution in a 500 ns MD simulation.

Supplementary Video S3. Binding profile of SmCesA2-PH on the PtdIns(3,4,5)P_3/POPC membrane in a 500 ns MD simulation.

Supplementary Video S4. Binding profile of SmCesA2-PH on the PtdIns(3,4)P_2/POPC membrane in a 500 ns MD simulation.

Supplementary Video S5. Binding profile of SmCesA2-PH on the POPC membrane in a 500 ns MD simulation.

Supplementary Video S6. Binding profile of SmCesA2-PH on the PtdIns/POPC membrane in a 500 ns MD simulation.

Appendix S1 Force field parameters of the head groups of phosphoinositides, obtained from CHARMM ParamChem web server

Ins

```
* Toppar stream file generated by
* CHARMM General Force Field (CGenFF) program version 0.9.7.1 beta
* For use with CGenFF version 2b8
*

read rtf card append
* Topologies generated by
* CHARMM General Force Field (CGenFF) program version 0.9.7.1 beta
*
36 1

! "penalty" is the highest penalty score of the associated parameters.
! Penalties lower than 10 indicate the analogy is fair; penalties between 10
! and 50 mean some basic validation is recommended; penalties higher than
! 50 indicate poor analogy and mandate extensive validation/optimization.

RESI INS          0.000 ! param penalty=     4.000 ; charge penalty=     0.000
GROUP                   ! CHARGE     CH_PENALTY
ATOM O2      OG311    -0.655 !       0.000
ATOM O3      OG311    -0.655 !       0.000
ATOM O4      OG311    -0.655 !       0.000
ATOM O5      OG311    -0.655 !       0.000
ATOM O6      OG311    -0.655 !       0.000
ATOM C11     CG311     0.146 !       0.000
ATOM C12     CG311     0.146 !       0.000
ATOM O12     OG311    -0.655 !       0.000
ATOM C13     CG311     0.146 !       0.000
ATOM C14     CG311     0.146 !       0.000
ATOM C15     CG311     0.146 !       0.000
ATOM C16     CG311     0.146 !       0.000
```

```
ATOM H1      HGP1     0.419 !     0.000
ATOM H2      HGP1     0.419 !     0.000
ATOM H3      HGP1     0.419 !     0.000
ATOM H4      HGP1     0.419 !     0.000
ATOM H5      HGP1     0.419 !     0.000
ATOM H6      HGA1     0.090 !     0.000
ATOM H7      HGA1     0.090 !     0.000
ATOM H8      HGP1     0.419 !     0.000
ATOM H9      HGA1     0.090 !     0.000
ATOM H10     HGA1     0.090 !     0.000
ATOM H11     HGA1     0.090 !     0.000
ATOM H12     HGA1     0.090 !     0.000

BOND O2   C12
BOND O3   C13
BOND O4   C14
BOND O5   C15
BOND O6   C16
BOND C11  C16
BOND C11  C12
BOND C11  O12
BOND C12  C13
BOND C13  C14
BOND C14  C15
BOND C15  C16
BOND O2   H1
BOND O3   H2
BOND O4   H3
BOND O5   H4
BOND O6   H5
BOND C11  H6
BOND C12  H7
BOND O12  H8
BOND C13  H9
BOND C14  H10
BOND C15  H11
BOND C16  H12

END

read param card flex append
* Parameters generated by analogy by
* CHARMM General Force Field (CGenFF) program version 0.9.7.1 beta
*

! Penalties lower than 10 indicate the analogy is fair; penalties between 10
! and 50 mean some basic validation is recommended; penalties higher than
! 50 indicate poor analogy and mandate extensive validation/optimization.

BONDS

ANGLES

DIHEDRALS
CG311  CG311  CG311  CG311     0.5000  4   180.00 ! INS , from CG311 CG311 CG311 CG321, penalty= 0.6
OG311  CG311  CG311  OG311     0.2000  3     0.00 ! INS , from OG311 CG311 CG321 OG311, penalty= 4

IMPROPERS

END
RETURN
```

Ins(1)P (PIP)

```
* Toppar stream file generated by
* CHARMM General Force Field (CGenFF) program version 0.9.7.1 beta
* For use with CGenFF version 2b8
*
```

```
read rtf card append
* Topologies generated by
* CHARMM General Force Field (CGenFF) program version 0.9.7.1 beta
*
36 1

! "penalty" is the highest penalty score of the associated parameters.
! Penalties lower than 10 indicate the analogy is fair; penalties between 10
! and 50 mean some basic validation is recommended; penalties higher than
! 50 indicate poor analogy and mandate extensive validation/optimization.

RESI PIP            -2.000 ! param penalty=   4.600 ; charge penalty=   2.132
GROUP                      ! CHARGE   CH_PENALTY
ATOM C11    CG311    0.146 !    2.087
ATOM C12    CG311    0.146 !    0.000
ATOM C13    CG311    0.146 !    2.087
ATOM C14    CG311    0.150 !    2.132
ATOM C15    CG311   -0.098 !    1.313
ATOM C16    CG311    0.150 !    2.132
ATOM O5     OG303   -0.399 !    1.313
ATOM P5     PG2      1.099 !    0.106
ATOM OP4    OG2P1   -0.900 !    0.000
ATOM OP5    OG2P1   -0.900 !    0.000
ATOM OP6    OG2P1   -0.900 !    0.000
ATOM O4     OG311   -0.655 !    0.300
ATOM O6     OG311   -0.655 !    0.300
ATOM O3     OG311   -0.655 !    0.000
ATOM O12    OG311   -0.655 !    0.000
ATOM O2     OG311   -0.655 !    0.000
ATOM H1     HGA1     0.090 !    0.000
ATOM H2     HGA1     0.090 !    0.000
ATOM H3     HGA1     0.090 !    0.000
ATOM H4     HGA1     0.090 !    0.300
ATOM H5     HGA1     0.090 !    0.000
ATOM H6     HGA1     0.090 !    0.300
ATOM H7     HGP1     0.419 !    0.000
ATOM H8     HGP1     0.419 !    0.000
ATOM H9     HGP1     0.419 !    0.000
ATOM H10    HGP1     0.419 !    0.000
ATOM H11    HGP1     0.419 !    0.000

BOND C11  C16
BOND C11  O12
BOND C11  C12
BOND C12  O2
BOND C12  C13
BOND C13  C14
BOND C13  O3
BOND C14  O4
BOND C14  C15
BOND C15  O5
BOND C15  C16
BOND C16  O6
BOND O5   P5
BOND P5   OP4
BOND P5   OP6
BOND P5   OP5
BOND C11  H1
BOND C12  H2
BOND C13  H3
BOND C14  H4
BOND C15  H5
BOND C16  H6
BOND O4   H7
BOND O6   H8
BOND O3   H9
BOND O12  H10
BOND O2   H11

END

read param card flex append
```

```
* Parameters generated by analogy by
* CHARMM General Force Field (CGenFF) program version 0.9.7.1 beta
*

! Penalties lower than 10 indicate the analogy is fair; penalties between 10
! and 50 mean some basic validation is recommended; penalties higher than
! 50 indicate poor analogy and mandate extensive validation/optimization.

BONDS

ANGLES
CG311    CG311    OG303      115.00        109.70 ! PIP , from CG331 CG311 OG303, penalty= 1.5

DIHEDRALS
CG311    CG311    CG311    CG311           0.5000    4      180.00 ! PIP , from CG311 CG311 CG311 CG321, penalty= 0.6
CG311    CG311    CG311    OG303           0.2000    3      180.00 ! PIP , from CG321 CG311 CG321 OG303, penalty= 4.6
OG303    CG311    CG311    OG311           0.2000    3        0.00 ! PIP , from OG311 CG311 CG321 OG303, penalty= 4
OG303    CG311    CG311    HGA1            0.1950    3        0.00 ! PIP , from HGA1 CG311 CG321 OG303, penalty= 4
OG311    CG311    CG311    OG311           0.2000    3        0.00 ! PIP , from OG311 CG311 CG321 OG311, penalty= 4
CG311    CG311    OG303    PG2             0.4000    1      180.00 ! PIP , from CG331 CG311 OG303 PG2, penalty= 1.5
CG311    CG311    OG303    PG2             0.3000    2        0.00 ! PIP , from CG331 CG311 OG303 PG2, penalty= 1.5
CG311    CG311    OG303    PG2             0.1000    3        0.00 ! PIP , from CG331 CG311 OG303 PG2, penalty= 1.5

IMPROPERS

END
RETURN
```

Ins(3,4)P_2 (PIP2)

```
* Toppar stream file generated by
* CHARMM General Force Field (CGenFF) program version 0.9.7.1 beta
* For use with CGenFF version 2b8
*

read rtf card append
* Topologies generated by
* CHARMM General Force Field (CGenFF) program version 0.9.7.1 beta
*
36 1

! "penalty" is the highest penalty score of the associated parameters.
! Penalties lower than 10 indicate the analogy is fair; penalties between 10
! and 50 mean some basic validation is recommended; penalties higher than
! 50 indicate poor analogy and mandate extensive validation/optimization.

RESI PIP2          -3.000 ! param penalty=    6.500 ; charge penalty=    2.986
GROUP                     ! CHARGE   CH_PENALTY
ATOM C11     CG311     0.146 !    2.087
ATOM C12     CG311     0.150 !    2.984
ATOM C13     CG311    -0.094 !    2.470
ATOM C14     CG311     0.003 !    2.514
ATOM C15     CG311     0.150 !    2.986
ATOM C16     CG311     0.146 !    2.087
ATOM O3      OG303    -0.399 !    1.278
ATOM P3      PG2       1.099 !    0.106
ATOM O33     OG2P1    -0.900 !    0.000
ATOM O32     OG2P1    -0.900 !    0.000
ATOM O31     OG2P1    -0.900 !    0.000
ATOM O2      OG311    -0.655 !    0.300
ATOM O4      OG303    -0.621 !    1.401
ATOM P4      PG1       1.499 !    0.526
ATOM O42     OG311    -0.670 !    0.268
ATOM O41     OG2P1    -0.823 !    0.030
ATOM O43     OG2P1    -0.823 !    0.030
ATOM O12     OG311    -0.655 !    0.000
ATOM O5      OG311    -0.655 !    0.300
ATOM O6      OG311    -0.655 !    0.000
ATOM H11     HGA1      0.090 !    0.000
```

```
ATOM H12       HGA1      0.090 !       0.300
ATOM H13       HGA1      0.090 !       0.300
ATOM H14       HGA1      0.090 !       0.304
ATOM H15       HGA1      0.090 !       0.300
ATOM H16       HGA1      0.090 !       0.000
ATOM HO2       HGP1      0.419 !       0.000
ATOM HO42      HGP1      0.341 !       0.000
ATOM HO12      HGP1      0.419 !       0.000
ATOM HO5       HGP1      0.419 !       0.000
ATOM HO6       HGP1      0.419 !       0.000

BOND C11    C12
BOND C11    C16
BOND C11    O12
BOND C11    H11
BOND C12    C13
BOND C12    O2
BOND C12    H12
BOND C13    C14
BOND C13    O3
BOND C13    H13
BOND C14    C15
BOND C14    O4
BOND C14    H14
BOND C15    C16
BOND C15    O5
BOND C15    H15
BOND C16    O6
BOND C16    H16
BOND O3     P3
BOND P3     O33
BOND P3     O32
BOND P3     O31
BOND O2     HO2
BOND O4     P4
BOND P4     O42
BOND P4     O41
BOND P4     O43
BOND O42    HO42
BOND O12    HO12
BOND O5     HO5
BOND O6     HO6

END

read param card flex append
* Parameters generated by analogy by
* CHARMM General Force Field (CGenFF) program version 0.9.7.1 beta
*

! Penalties lower than 10 indicate the analogy is fair; penalties between 10
! and 50 mean some basic validation is recommended; penalties higher than
! 50 indicate poor analogy and mandate extensive validation/optimization.

BONDS

ANGLES
CG311    CG311    OG303     115.00      109.70 ! PIP2 , from CG331 CG311 OG303, penalty= 1.5
CG311    OG303    PG1        20.00      120.00     35.00     2.33000 ! PIP2 , from CG321 OG303 PG1, penalty= 0.6

DIHEDRALS
CG311    CG311    CG311    CG311       0.5000   4     180.00 ! PIP2 , from CG311 CG311 CG311 CG321, penalty= 0.6
CG311    CG311    CG311    OG303       0.2000   3     180.00 ! PIP2 , from CG321 CG311 CG321 OG303, penalty= 4.6
OG303    CG311    CG311    OG303       0.2000   3       0.00 ! PIP2 , from OG302 CG311 CG321 OG303, penalty= 6.5
OG303    CG311    CG311    OG311       0.2000   3       0.00 ! PIP2 , from OG311 CG311 CG321 OG303, penalty= 4
OG303    CG311    CG311    HGA1        0.1950   3       0.00 ! PIP2 , from HGA1 CG311 CG321 OG303, penalty= 4
OG311    CG311    CG311    OG311       0.2000   3       0.00 ! PIP2 , from OG311 CG311 CG321 OG311, penalty= 4
CG311    CG311    OG303    PG1         0.4000   1     180.00 ! PIP2 , from CG331 CG311 OG303 PG2, penalty= 2.5
CG311    CG311    OG303    PG1         0.3000   2       0.00 ! PIP2 , from CG331 CG311 OG303 PG2, penalty= 2.5
CG311    CG311    OG303    PG1         0.1000   3       0.00 ! PIP2 , from CG331 CG311 OG303 PG2, penalty= 2.5
CG311    CG311    OG303    PG2         0.4000   1     180.00 ! PIP2 , from CG331 CG311 OG303 PG2, penalty= 1.5
CG311    CG311    OG303    PG2         0.3000   2       0.00 ! PIP2 , from CG331 CG311 OG303 PG2, penalty= 1.5
```

```
CG311   CG311   OG303   PG2      0.1000  3    0.00 ! PIP2 , from CG331 CG311 OG303 PG2, penalty= 1.5
HGA1    CG311   OG303   PG1      0.0000  3    0.00 ! PIP2 , from HGA1 CG311 OG303 PG2, penalty= 1
CG311   OG303   PG1     OG2P1    0.1000  3    0.00 ! PIP2 , from CG321 OG303 PG1 OG2P1, penalty= 0.6
CG311   OG303   PG1     OG311    0.9500  2    0.00 ! PIP2 , from CG321 OG303 PG1 OG311, penalty= 0.6
CG311   OG303   PG1     OG311    0.5000  3    0.00 ! PIP2 , from CG321 OG303 PG1 OG311, penalty= 0.6

IMPROPERS

END
RETURN
```

Ins(3,4,5)P_3 (PIP3)

```
* Toppar stream file generated by
* CHARMM General Force Field (CGenFF) program version 0.9.7.1 beta
* For use with CGenFF version 2b8
*

read rtf card append
* Topologies generated by
* CHARMM General Force Field (CGenFF) program version 0.9.7.1 beta
*
36 1

! "penalty" is the highest penalty score of the associated parameters.
! Penalties lower than 10 indicate the analogy is fair; penalties between 10
! and 50 mean some basic validation is recommended; penalties higher than
! 50 indicate poor analogy and mandate extensive validation/optimization.

RESI PIP3          -5.000 ! param penalty=   6.500 ; charge penalty=   3.268
GROUP                ! CHARGE   CH_PENALTY
ATOM P3     PG1      1.499 !    0.526
ATOM O31    OG2P1   -0.823 !    0.030
ATOM O32    OG2P1   -0.823 !    0.030
ATOM O3     OG303   -0.621 !    1.401
ATOM C13    CG311    0.003 !    3.268
ATOM C12    CG311    0.150 !    2.986
ATOM C11    CG311    0.146 !    2.952
ATOM C16    CG311    0.150 !    2.984
ATOM C15    CG311   -0.094 !    3.232
ATOM C14    CG311   -0.090 !    3.235
ATOM O2     OG311   -0.655 !    0.300
ATOM O12    OG311   -0.655 !    0.000
ATOM O6     OG311   -0.655 !    0.300
ATOM O5     OG303   -0.399 !    1.278
ATOM P5     PG2      1.099 !    0.106
ATOM O51    OG2P1   -0.900 !    0.000
ATOM O53    OG2P1   -0.900 !    0.000
ATOM O52    OG2P1   -0.900 !    0.000
ATOM O4     OG303   -0.399 !    1.242
ATOM P4     PG2      1.099 !    0.106
ATOM O41    OG2P1   -0.900 !    0.000
ATOM O43    OG2P1   -0.900 !    0.000
ATOM O42    OG2P1   -0.900 !    0.000
ATOM O33    OG311   -0.670 !    0.268
ATOM H13    HGA1     0.090 !    0.304
ATOM H12    HGA1     0.090 !    0.300
ATOM H11    HGA1     0.090 !    0.000
ATOM H16    HGA1     0.090 !    0.300
ATOM H15    HGA1     0.090 !    0.300
ATOM H14    HGA1     0.090 !    0.424
ATOM HO2    HGP1     0.419 !    0.000
ATOM HO12   HGP1     0.419 !    0.000
ATOM HO6    HGP1     0.419 !    0.000
ATOM HO33   HGP1     0.341 !    0.000

BOND P3   O31
BOND P3   O32
BOND P3   O3
```

```
BOND P3      O33
BOND O3      C13
BOND C13     C12
BOND C13     C14
BOND C13     H13
BOND C12     C11
BOND C12     O2
BOND C12     H12
BOND C11     C16
BOND C11     O12
BOND C11     H11
BOND C16     C15
BOND C16     O6
BOND C16     H16
BOND C15     C14
BOND C15     O5
BOND C15     H15
BOND C14     O4
BOND C14     H14
BOND O2      HO2
BOND O12     HO12
BOND O6      HO6
BOND O5      P5
BOND P5      O51
BOND P5      O53
BOND P5      O52
BOND O4      P4
BOND P4      O41
BOND P4      O43
BOND P4      O42
BOND O33     HO33

END

read param card flex append
* Parameters generated by analogy by
* CHARMM General Force Field (CGenFF) program version 0.9.7.1 beta
*

! Penalties lower than 10 indicate the analogy is fair; penalties between 10
! and 50 mean some basic validation is recommended; penalties higher than
! 50 indicate poor analogy and mandate extensive validation/optimization.

BONDS

ANGLES
CG311    CG311    OG303      115.00       109.70 ! PIP3 , from CG331 CG311 OG303, penalty= 1.5
CG311    OG303    PG1         20.00       120.00      35.00      2.33000 ! PIP3 , from CG321 OG303 PG1, penalty= 0.6

DIHEDRALS
CG311    CG311    CG311    CG311        0.5000    4     180.00 ! PIP3 , from CG311 CG311 CG311 CG321, penalty= 0.6
CG311    CG311    CG311    OG303        0.2000    3     180.00 ! PIP3 , from CG321 CG311 CG321 OG303, penalty= 4.6
OG303    CG311    CG311    OG303        0.2000    3       0.00 ! PIP3 , from OG302 CG311 CG321 OG303, penalty= 6.5
OG303    CG311    CG311    OG311        0.2000    3       0.00 ! PIP3 , from OG311 CG311 CG321 OG303, penalty= 4
OG303    CG311    CG311    HGA1         0.1950    3       0.00 ! PIP3 , from HGA1 CG311 CG321 OG303, penalty= 4
OG311    CG311    CG311    OG311        0.2000    3       0.00 ! PIP3 , from OG311 CG311 CG321 OG311, penalty= 4
CG311    CG311    OG303    PG1          0.4000    1     180.00 ! PIP3 , from CG331 CG311 OG303 PG2, penalty= 2.5
CG311    CG311    OG303    PG1          0.3000    2       0.00 ! PIP3 , from CG331 CG311 OG303 PG2, penalty= 2.5
CG311    CG311    OG303    PG1          0.1000    3       0.00 ! PIP3 , from CG331 CG311 OG303 PG2, penalty= 2.5
CG311    CG311    OG303    PG2          0.4000    1     180.00 ! PIP3 , from CG331 CG311 OG303 PG2, penalty= 1.5
CG311    CG311    OG303    PG2          0.3000    2       0.00 ! PIP3 , from CG331 CG311 OG303 PG2, penalty= 1.5
CG311    CG311    OG303    PG2          0.1000    3       0.00 ! PIP3 , from CG331 CG311 OG303 PG2, penalty= 1.5
HGA1     CG311    OG303    PG1          0.0000    3       0.00 ! PIP3 , from HGA1 CG311 OG303 PG2, penalty= 1
CG311    OG303    PG1      OG2P1        0.1000    3       0.00 ! PIP3 , from CG321 OG303 PG1 OG2P1, penalty= 0.6
CG311    OG303    PG1      OG311        0.9500    2       0.00 ! PIP3 , from CG321 OG303 PG1 OG311, penalty= 0.6
CG311    OG303    PG1      OG311        0.5000    3       0.00 ! PIP3 , from CG321 OG303 PG1 OG311, penalty= 0.6

IMPROPERS

END
RETURN
```
